# New specimens of *Baurutitan britoi* and a taxonomic reassessment of the titanosaur dinosaur fauna (Sauropoda) from the Serra da Galga Formation (Late Cretaceous) of Brazil

**DOI:** 10.7717/peerj.14333

**Published:** 2022-11-15

**Authors:** Julian C. G. Silva Junior, Agustín G. Martinelli, Thiago S. Marinho, João Ismael da Silva, Max C. Langer

**Affiliations:** 1 Laboratório de Paleontologia de Ribeirão Preto, Faculdade de Filosofia, Ciências e Letras de Ribeirão Preto, Universidade de São Paulo, Ribeirão Preto, São Paulo, Brazil; 2 Sección Paleontología de Vertebrados, Museo Argentino de Ciencias Naturales “Bernardino Rivadavia”, Buenos Aires, Buenos Aires, Argentina; 3 Pró-Reitoria de Extensão Universitária, Universidade Federal do Triangulo Mineiro, Centro de Pesquisas Paleontológicas L. I. Price, Complexo Cultural e Científico Peirópolis, Uberaba, Minas Gerais, Brazil; 4 Departamento de Ciências Biológicas, Instituto de Ciências Exatas, Naturais e Educação, Universidade Federal do Triângulo Mineiro, Uberaba, Minas Gerais, Brazil; 5 Prefeitura Municipal de Uberaba, Fundação Cultural de Uberaba, Uberaba, Minas Gerais, Brazil

**Keywords:** Titanosaur, Sauropoda, Late Cretaceous, Brazil, Reassement

## Abstract

The description of new titanosaur specimens unearthed from deposits of the Serra da Galga Formation (Bauru Group, Late Cretaceous) at the BR-262 site, near Peirópolis (Uberaba, Minas Gerais State, Brazil), sheds light on the taxonomy of two taxa previously known from the same area and geological unit: *Baurutitan britoi* and *Trigonosaurus pricei*. A comparative revision indicates that *T. pricei* represents a junior synonym of *Ba. britoi*, and that the BR-262 specimens belong to that latter species. The information provided by the new specimens also revealed that the paratype of *T. pricei* (MCT 1719-R), a caudal vertebral series, actually represents a new taxon, named here as *Caieiria allocaudata* gen. et sp. nov.

## Introduction

*Titanosauria* currently represents the most species-rich dinosaur clade in the Brazilian Cretaceous (Bittencourt & Langer, 2011; Ghilardi et al., 2016; Carvalho et al., 2017; Bandeira et al., 2018), with numerous records coming from the Serra da Galga Formation (Bauru Group, Bauru Basin) in the surroundings of Uberaba, Minas Gerais State (Candeiro et al., 2006; Martinelli & Teixeira, 2015). Field work carried-out in that area, from the late 1940’s to the 1960’s, by the Brazilian paleontologist Llewellyn Ivor Price, were especially productive (Campos & Kellner, 1999), followed by systemic excavations conducted by the Centro de Pesquisas Paleontológicas Llewellyn Ivor Price (CPPLIP) and Museu dos Dinossauros since the beginning of the 1990s.

Price was responsible for unearthing a remarkable set of titanosaur remains from the quarry known as “Caieira”, a site he called “Ponto 1”, located less than 2 km from the town of Peirópolis and about 20 km east of Uberaba. The material was later assigned to supposedly individual specimens known as Series A, B, and C (Powell, 1987, 2003; Bertini, 1993; Campos & Kellner, 1999). Series A (MCT 1487-R) consists of 12 cervical and three anterior trunk vertebrae. It was only partially described by Powell (1987, 2003) and until recently remained unassigned to any particular taxon. Silva Junior et al. (2019) suggested its referral to *Uberabatitan ribeiroi*, another species from the Serra da Galga Formation, the holotype of which was unearthed from the “BR-050 Km 153” locality, about 40 km from “Caieira”.

Series B (MCT 1488-R) is one of the best-preserved titanosaurs recorded in the area, consisting of five cervical and ten trunk vertebrae, the sacrum, and one ilium. Powell (1987) considered a set of 10 caudal vertebrae (MCT 1719-R) as possibly articulated, and assigned it to Series B. This association was questioned by Campos & Kellner (1999, p. 22); according to whom: “Price separated the caudal vertebrae of Series B from the pelvis and, as far as known, never regarded them as belonging to the same individual”. However, in proposing a new species, *Trigonosaurus pricei*, based on MCT 1488-R, Campos et al. (2005) assigned the caudal sequence MCT 1719-R as its paratype. In support of the referral of MCT 1719-R tail vertebrae to *T. pricei*, Campos et al. (2005, p. 3) stated that: “their size is compatible with the sacral elements and therefore we cannot preclude the possibility that they belong to the same individual represented by MCT 1488-R, as has been apparently assumed by Price”. It is, therefore, controversial whether or not Price associated MCT 1719-R with MCT 1488-R.

Finally, Series C (MCT 1490-R) consists of the last sacral and eighteen caudal vertebrae with 15 articulated chevrons. This specimen represents the holotype of *Baurutitan britoi*, as proposed by Kellner, Campos & Trotta (2005). Owing to the completeness of the sequence and the presence of the first caudal vertebra, *Ba. britoi* has been used in studies focusing on titanosaur tail musculature and anatomy (*e.g*., Gallina & Otero, 2009; Ibiricu, Lamanna & Lacovara, 2014).

**Figure 1:**
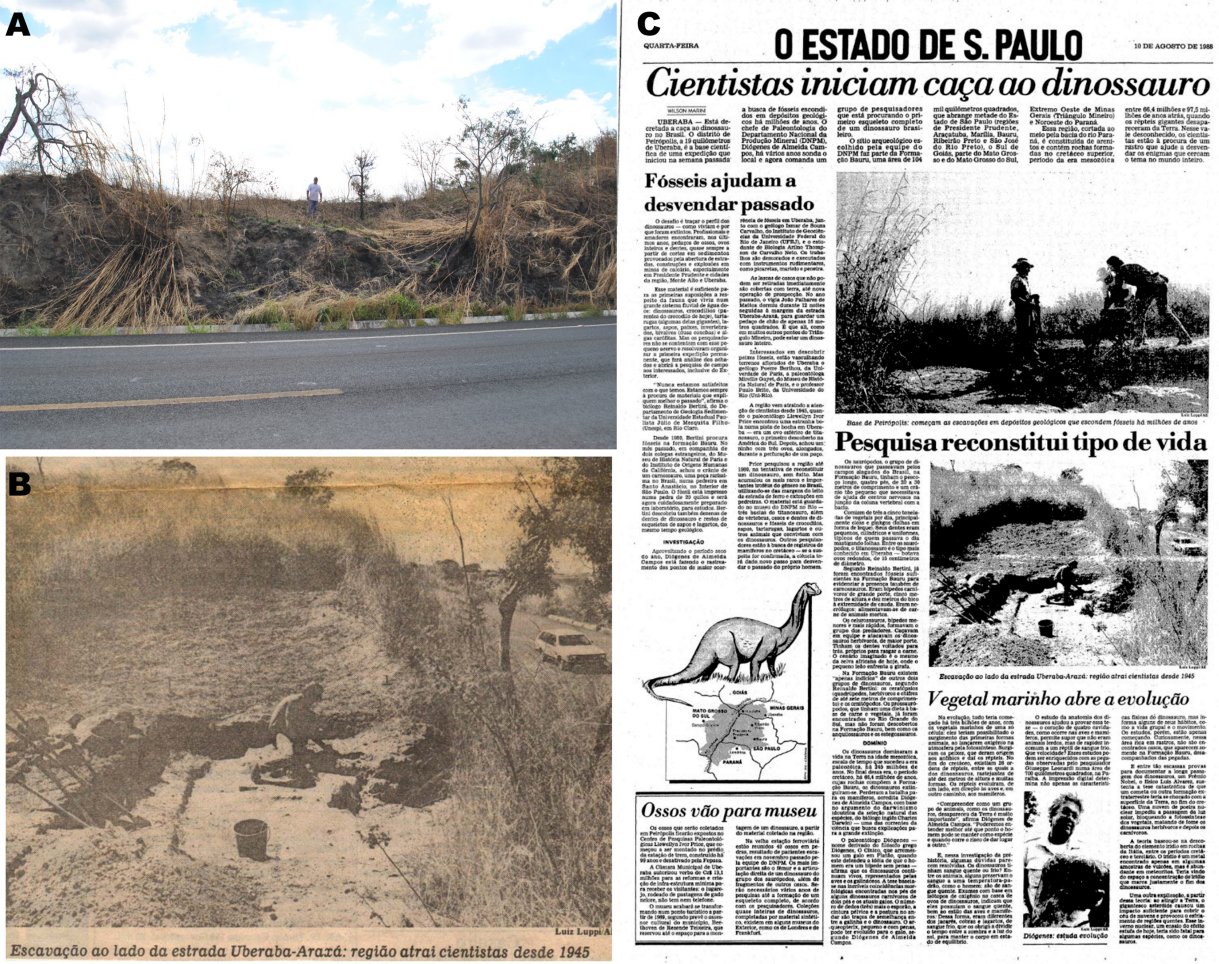
(A) “Rodovia” quarry in 2012 (photo by AGM). (B and C) News article depicting the field works in 1988 (from the archives of Beethoven Teixeira).

Another site in which Price had been working was “Point 6” or “Rodovia”, located about 1.5 km east of Peirópolis, in the northern slope of BR-262 highway (Fig. 1). Field work during the 1980s and 1990s resulted in the recovery of titanosaur bones, including cervical, trunk, and caudal vertebrae, plus appendicular elements, all in close association. A preliminary report by Martinelli et al. (2014) indicated that the trunk vertebrae resemble those of *T. pricei*, whereas the caudal vertebrae resemble those of *Ba. britoi*. Here we provide a full anatomical description of all titanosaur specimens collected at “Rodovia”, which are housed at CPPLIP. This led to a taxonomic revision of both *T. pricei* and *Ba. britoi*, as well as to the reassessment of MCT 1719-R.

### Geological settings

**Figure 2:**
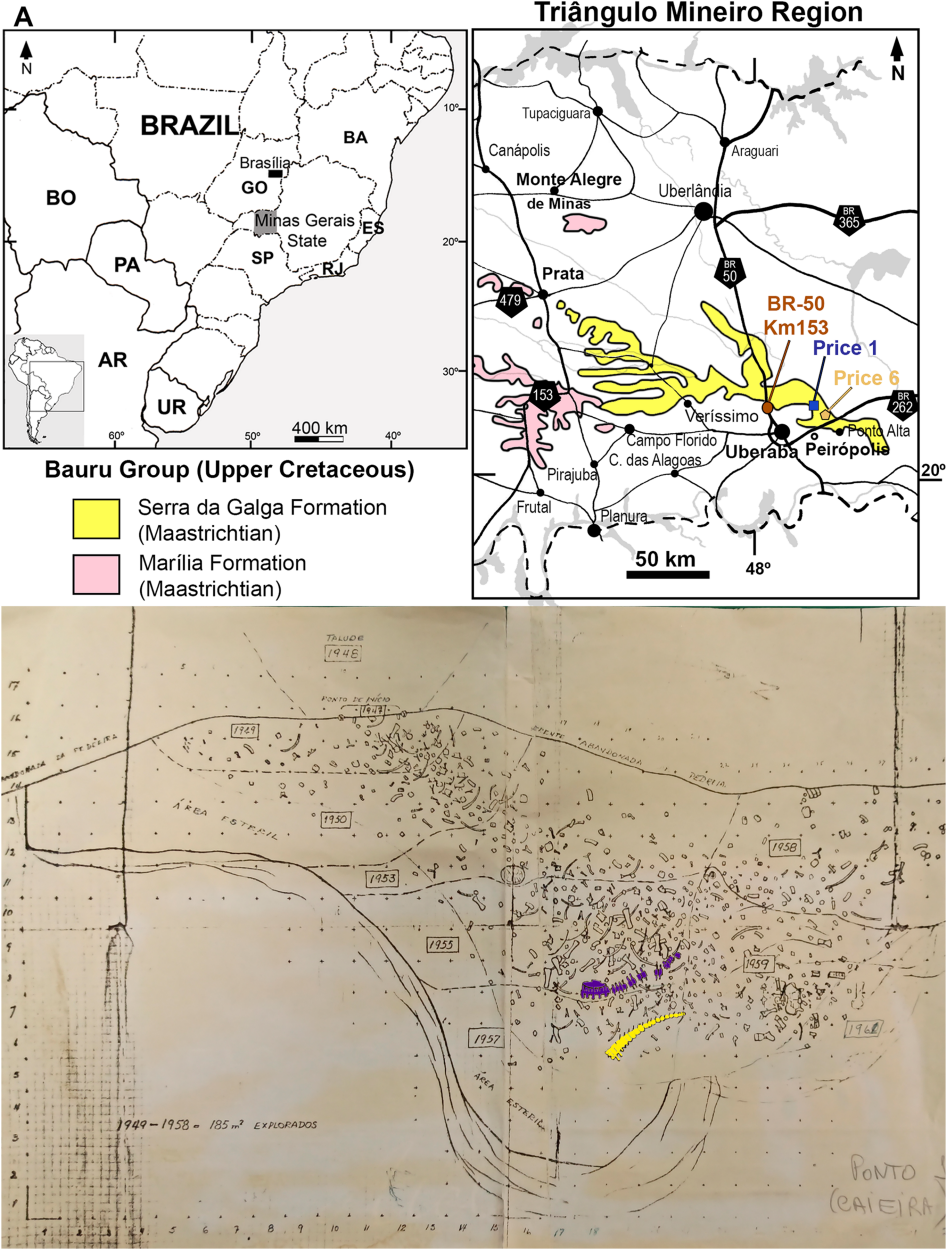
(A) Map of the Bauru Basin detailing the Uberaba region. (B) Map of “Ponto 1” quarry made by Price, detailing positions of Series B (Purple) and C (Yellow). It is noteworthy that MCT 1719-R cannot be located on the map (From the archives of CPPLIP).

The “Rodovia” quarry (here termed as BR-262 site) is located about 1.5 km southeast of Price’s “Ponto 1” (Fig. 2) as part of a series of outcrops located along the Veadinho Hills (*i.e*., “Serra do Veadinho”; Campos & Kellner, 1999; Martinelli et al., 2015). The sandstone layers exposed at the site are equivalent to the most fossiliferous levels of “Ponto 1” (Campos & Kellner, 1999; Martinelli et al., 2015, 2019; Soares et al., 2021) and correspond to the Serra da Galga Formation, Bauru Group, with a Maastrichtian age (Fernandes & Ribeiro, 2015; Martinelli et al., 2019; Soares et al., 2020, 2021). The detailed geological setting of the Serra da Galga Formation at the Veadinho Hills was described by Soares et al. (2020, 2021). The holotypes of *T. pricei* and *Ba. britoi*, the referred specimen MCT 1719-R, and the new material here described were unearthed at the base of their respective outcrops from structureless medium- and fine-grained sandstone, which are part of a distributive fluvial system with overall direction of flow to the NNW, developed under the influence of a semiarid climate regime (Soares et al., 2020, 2021).

## Materials and Methods

The specimen described here, as well as those used for comparisons, belong to public collections and were examined with the explicit permission of appropriate curators and/or collection managers. We followed all Brazilian regulations for fossil collection.

We employ the nomenclature proposed by Wilson (1999, 2012) and Wilson et al. (2011) to describe the laminae and fossae of titanosaur vertebrae. For muscle-related structures we follow Borsuk-Białynicka (1977) and Voegele et al. (2020, 2021).

Following article 6, recommendation 6.1A, from the PhyloCode (Cantino & De Queiroz, 2020), all clades established under that code are italicized.

The electronic version of this article in Portable Document Format (PDF) will represent a published work according to the International Commission on Zoological Nomenclature (ICZN), and hence the new names contained in the electronic version are effectively published under that Code from the electronic edition alone. This published work and the nomenclatural acts it contains have been registered in ZooBank, the online registration system for the ICZN. The ZooBank LSIDs (Life Science Identifiers) can be resolved and the associated information viewed through any standard web browser by appending the LSID to the prefix http://zoobank.org/. The LSID for this publication is: urn:lsid:zoobank.org:pub:28423C0B-A3E2-4ABF-8751-2E3A8FA98D4A. The online version of this work is archived and available from the following digital repositories: PeerJ, PubMed Central SCIE and CLOCKSS.

### Phylogenetic analysis

In order to assess the phylogenetic position of the species revised here, we performed a couple of phylogenetic analyses using a modified version of the Silva Junior et al. (2022) dataset, which is itself modified from Hechenleitner et al. (2020) (Files S2 and S3), with the addition of MCT 1719-R and the BR-262 specimens as new operational taxonomic units (OTUs). For a second iteration, the BR-262 specimens coding was combined with *Baurutitan britoi* and *Trigonosaurus pricei* as a single OTU, with both states kept for polymorphic characters. The analyses were conducted in TNT 1.5 (Goloboff & Catalano, 2016) with equal weighting of characters and tree bisection and reconnection (TBR) as the branch swapping algorithm, hold established as 50, 5,000 replicates, and random seeds as ‘0’. A total of 24 characters were considered as ordered (14, 61, 100, 102, 109, 115, 127, 132, 135, 136, 167, 180, 196, 257, 260, 277, 278, 279, 280, 300, 304, 347, 353, 355). The data scores are detailed in File S1.

## Description

Aside from a disproportionally large humerus (CPPLIP-263), all other BR-262 remains are compatible in size so they could represent a single individual. Moreover, we found no *a priori* anatomical differences among the elements indicating the presence of more than one taxon in the quarry. A direct comparison to the *U. ribeiroi* bonebed (Salgado & De Souza Carvalho, 2008; Silva Junior et al., 2019) can be useful. Even with the presence of individuals of different sizes and ontogenetic stages, the specimens of *U. ribeiroi* share several anatomical traits, such as the laminar patterns of the cervical vertebrae, a low degree of pneumatization in the trunk vertebrae, caudal neural spines that vary from vertically oriented to only slightly inclined anteriorly, and chevrons with dorsally open haemal canals and mediolaterally flattened distal processes. An equivalent congruent anatomy is seen within the BR-262 specimens. The middle cervical vertebrae share a robust postzygodiapophyseal lamina and a low neural spine, whereas the trunk vertebrae are highly pneumatized and bear posteriorly inclined neural spines. Posteriorly inclined neural spines are also present in all caudal vertebrae and the chevrons share dorsally closed haemal canals and robust proximal processes.

### Axial skeleton

*Cervical vertebrae*. Four sauropod cervical vertebrae (CPPLIP-035, CPPLIP-039, CPPLIP-040 and CPPLIP-049) were recovered from BR-262 site. Based on traits such as the width of neural canals and height of neural spines, the four elements were assigned to their respective regions of the neck.

**Figure 3:**
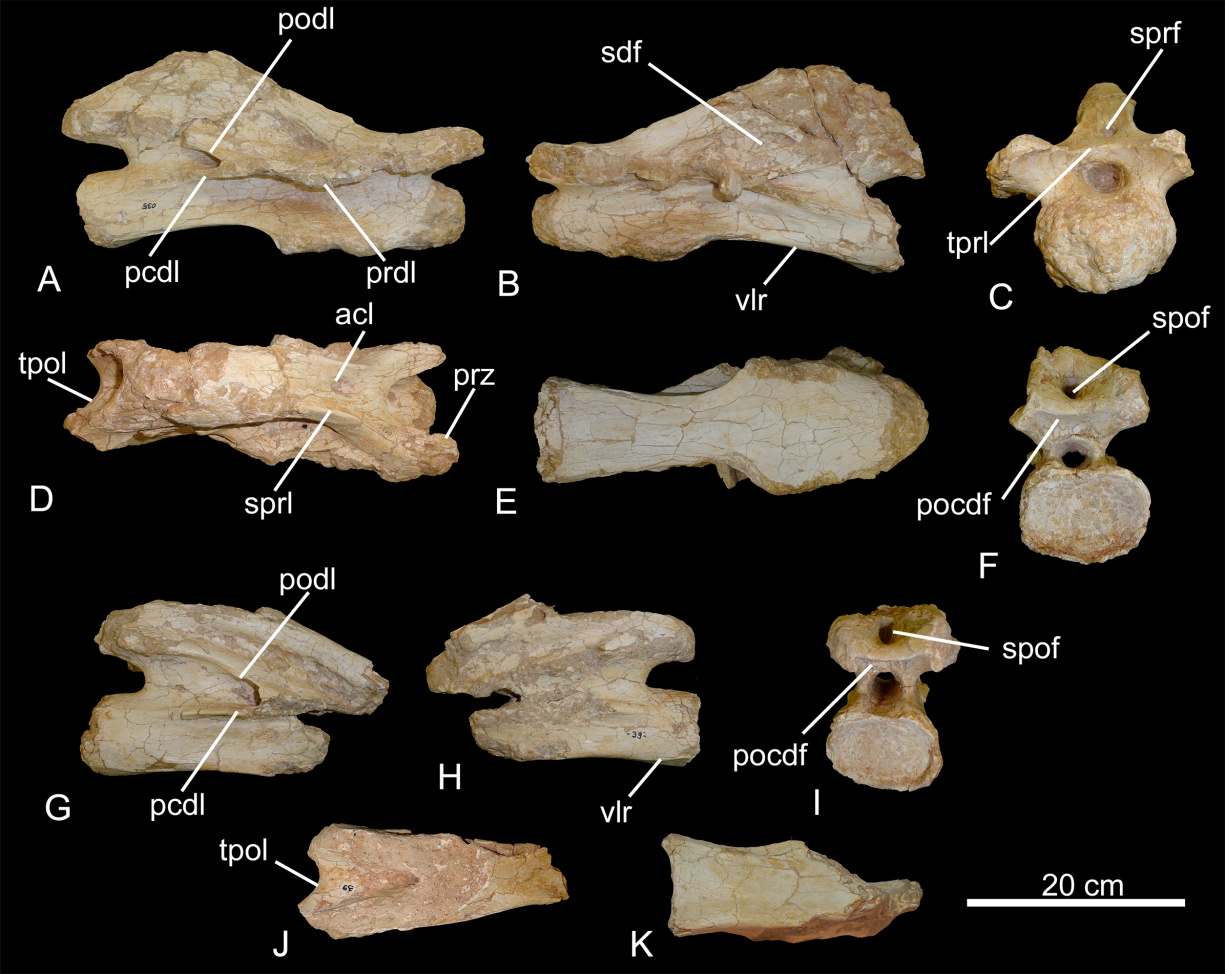
Middle cervical vertebrae of the BR-262 specimens. CPPLIP-035 in (A) right lateral; (B) left lateral; (C) anterior; (D) dorsal; (E) ventral and (F) posterior views. CPPLIP-039 in (G) right lateral; (H) left lateral; (I) posterior; (J) dorsal and (K) ventral views. Abbreviations: acl, accessory lamina; pcdl, posterior centrodiapophyseal lamina; pocdf, postzygapophyseal centrodiapophyseal fossa; podl, postzygodiapophyseal lamina; prdl, prezygodiapophyseal lamina; sdf, spinodiapophyseal fossa; spof, spinopostzygapophyseal fossa; sprl, spinoprezygapophyseal lamina; tpol, interpostzygapophyseal lamina; tprl, interprezygapophyseal lamina; vlr, ventrolateral ridge.

CPPLIP-035 and 039 (middle cervical vertebrae; Fig. 3). These two vertebrae possess a similar anatomy, but have different states of preservation. CPPLIP-039 lacks its anterior half, the distal portion of the neural spine, and all laminae from the left side. CPPLIP-035 lacks the parapophyses and diapophyses, with the postzygapophyses and laminae slightly better preserved on the right side.

The centra are anteroposteriorly elongated and dorsoventrally shallow. CPPLIP-035 has an aEI (average elongation index; Chure et al., 2010) of 3.4. The anterior margins of the condyles lie at the same anteroposterior level as those of the prezygapophyses. The cotyles are wider than deep, circular in posterior view, and extend as posteriorly as the interpostzygapophyseal laminae. Ventrolateral ridges form thin laminae that project laterally from the ventral margins of the centra. The ventral surfaces of the centra are slightly concave in both lateral and anterior views. The pneumatic fossae are deep, extending from the posterior portion of the condyles to the dorsal contact between the postzygodiapophyseal and the posterior centrodiapophyseal laminae.

In lateral view, the prezygapophyses extend anterodorsally, with the articular facets positioned immediately dorsal to the condyles, facing medially. They connect posteromedially with the interprezygapophyseal laminae, which extend until the anterior margin of the neural canal. The spinoprezygapophyseal laminae delimit the spinoprezygapophyseal fossa laterally, the spinodiapophyseal fossae dorsally, and reach the distal tip of the neural spines. The neural spines are triangular in lateral view, displaced posteriorly and each possess a ‘bulbous’, *i.e*., mediolaterally expanded, apex. They are anteriorly limited by the spinoprezygapophyseal fossae, which are shallow and perforated by small depressions, and laterally delimitated by accessory laminae in CPPLIP-035. The spinopostzygapophyseal laminae are not preserved.

The diapophyses and parapophyses are poorly preserved and lay posterior to the condyles. The diapophyses are connected to the centra *via* the posterior centrodiapophyseal laminae, situated below the spinodiapophyseal fossae, and connected to the prezygapophyses by the prezygodiapophyseal laminae. They reach posteriorly the contact between the postzygodiapophyseal and posterior centrodiapophyseal laminae. The postzygapophyses are not preserved, but were connected to one another *via* the interpostzygapophyseal laminae, which have almost the same breadth as the neural canal, and separate the spinopostzygapophyseal fossa from the postzygapophyseal centrodiapophyseal fossae. Each of the former fossae is also pierced by a large depression, which is not surrounded by accessory laminae.

**Figure 4:**
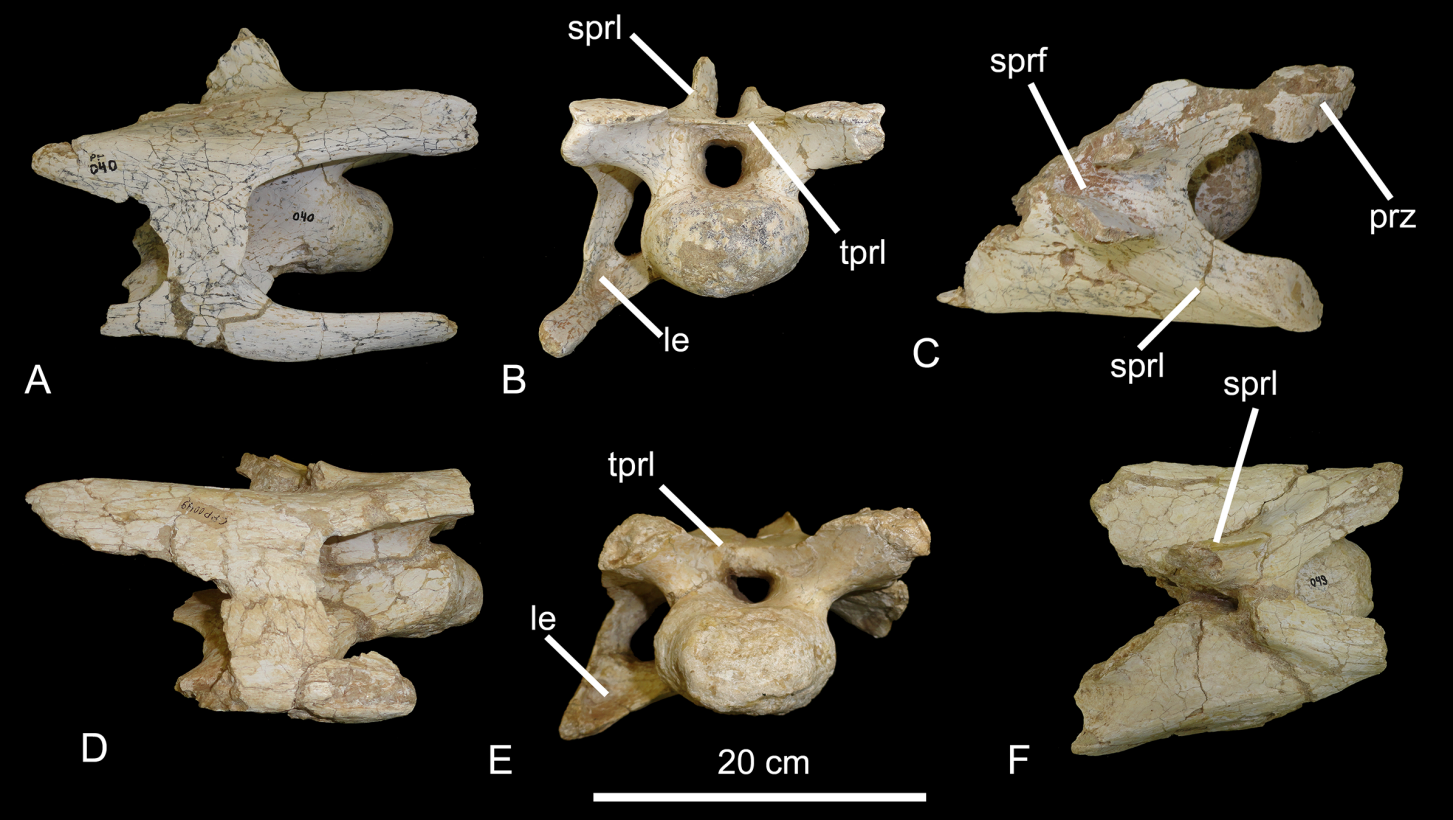
Posterior cervical vertebrae of the BR-262 specimens. CPPLIP-040 in (A) right lateral; (B) anterior and (C) dorsal views. CPPLIP-049 in (D) right lateral; (E) anterior and (F) dorsal views. Abbreviations: eprl, epipophyseal-prezygapophyseal laminae; le, longitudinal excavation; prz, prezygapophyses; sprl, spinoprezygapophyseal lamina; tprl, interprezygapophyseal lamina.

CPPLIP-040 and 049 (posterior cervical vertebrae, Fig. 4). These two vertebrae possess similar anatomy and preservation, with only their anteriormost portions and prezygapophyses preserved.

On the anterior portion of the centra that are preserved, shallow pneumatic fossae are visible and the lateral surfaces are slightly concave anteroposteriorly. The prezygapophyses do not overhang the centrum, and extend anterodorsally, with the articular facets facing mediodorsally. The prezygapophyses are connected posteromedially by the interprezygapophyseal lamina, which extends anteriorly in CPPLIP-040. In CPPLIP-049, only small anterior portions of the spinoprezygapophyseal laminae are preserved, whereas larger portions are preserved in CPPLIP-040. The spinoprezygapophyseal laminae delimit deep spinoprezygapophyseal fossae laterally. Laterally, diapophyses and parapophyses are preserved only on the right side. The diapophyses lay posterior to the condyles and the parapophyses are short and slightly bent downwards, with shallow excavations dorsally.

**Figure 5:**
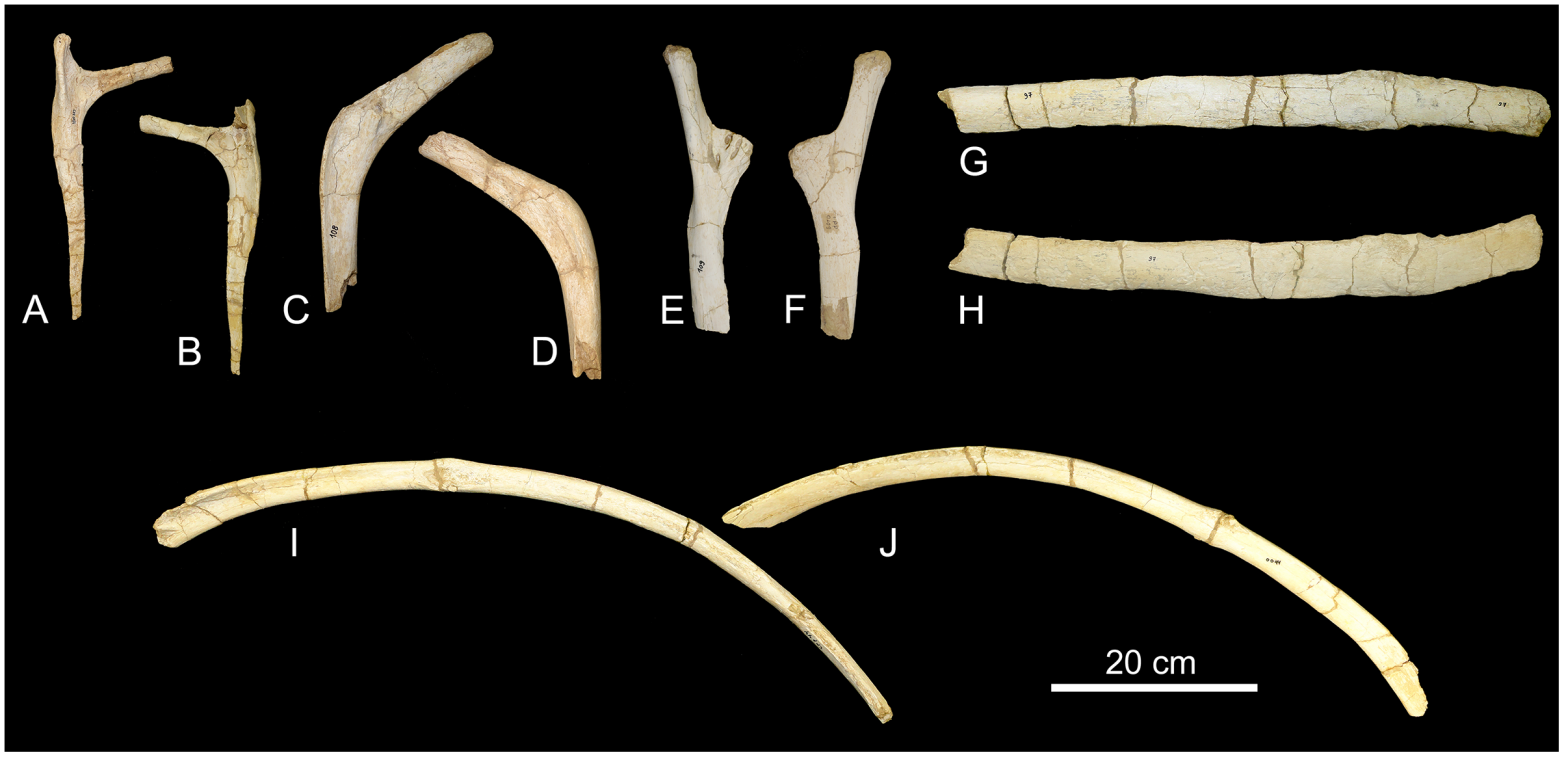
Cervical and trunk ribs of the BR-262 specimens. CPPLIP-014 (cervical rib) in (A) lateral and (B) medial views. CPPLIP-108 (trunk rib) in (C) anterior and (D) posterior views. CPPLIP-109 (trunk rib) in (E) lateral and (F) medial views. CPPLIP-097 (trunk rib) in (G) dorsal and (H) ventral views. CPPLIP-044 (trunk rib) in (I) anterior and (J) posterior views.

*Cervical ribs*. Two partially preserved, isolated cervical ribs (CPPLIP-014 and CPPLIP-109; Fig. 5) were recovered from BR-262. They are gracile elements, mainly corresponding to mediolaterally flattened laminae, each with a shallow dorsal concavity on the proximal portion. CPPLIP-109 has several small foramina on its most anterior portion. The tuberculum of CPPLIP-014 forms a thin lamina, whereas that of CPPLIP-109 is more robust, but both project dorsoventrally. Their capitula are not preserved.

*Trunk vertebrae*. Eight sauropod trunk elements were recovered from BR-262: seven complete vertebrae (CPPLIP-036, CPPLIP-037, CPPLIP-043, CPPLIP-103, CPPLIP-110, CPPLIP-111 and CPPLIP-458) and a posterior neural spine (CPPLIP-043). The location of the eight elements along the trunk was identified based on the development of the pre- and postzygapophyses and the position of parapophyses and diapophyses.

**Figure 6:**
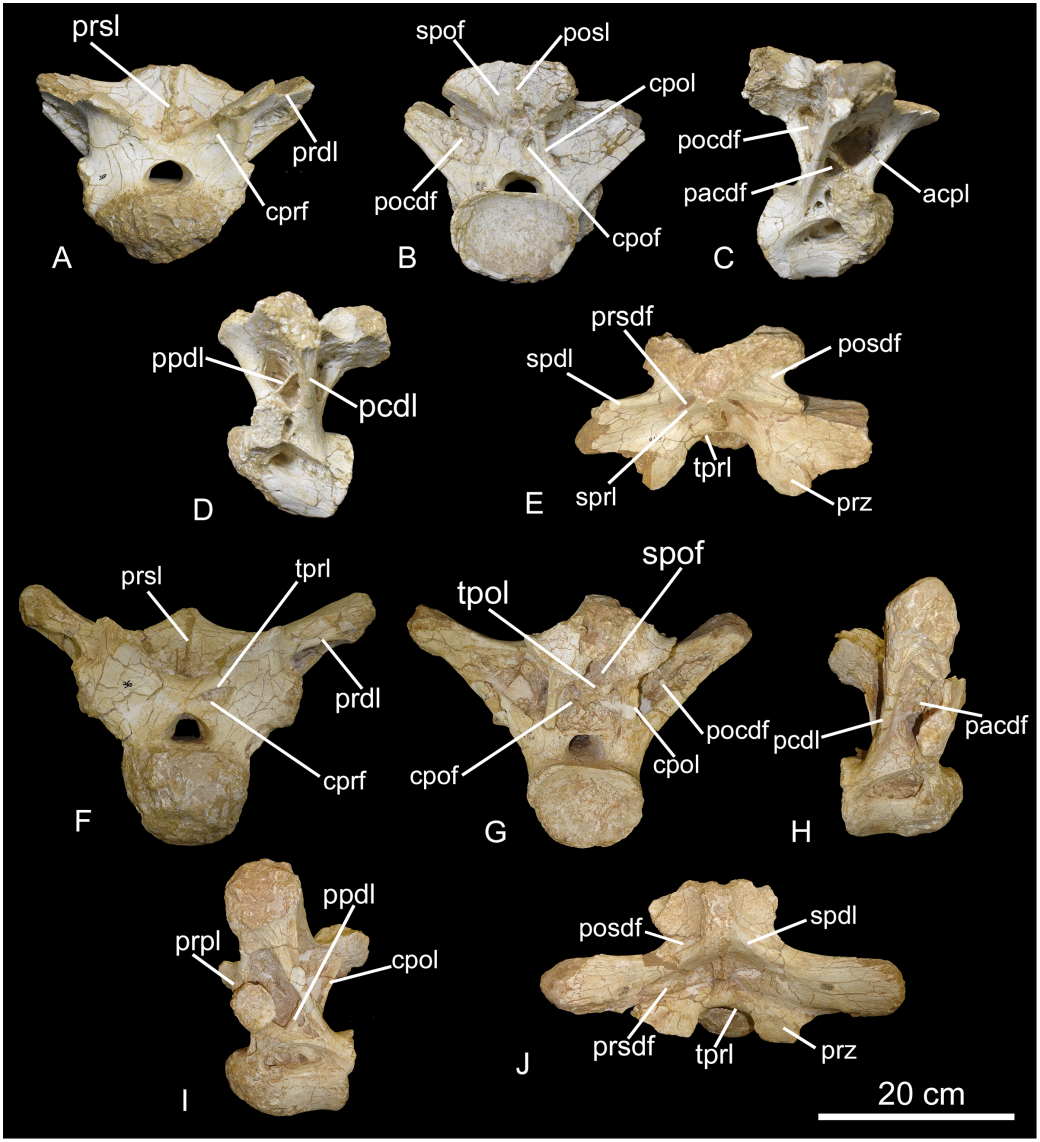
Anterior trunk vertebrae of the BR-262 specimens. CPPLIP-110 in (A) anterior; (B) posterior; (C) right lateral; (D) left lateral and (E) dorsal views. CPPLIP-036 in (F) anterior; (G) posterior; (H) right lateral; (I) left lateral and (J) dorsal views. Abbreviations: acpl, anterior centroparapophyseal lamina; cdf, centrodiapophyseal fossa; cpof, centropostzygapophyseal fossa; cpol, centropostzygapophyseal lamina; cprf, centroprezygapophyseal fossa; pacdf, parapophyseal centrodiapophyseal fossa; pcdl, posterior centrodiapophyseal lamina; pocdf, postzygapophyseal centrodiapophyseal fossa; ppdl, paradiapophyseal lamina; posdf, postzygapophyseal spinodiapophyseal fossa; prpl, prezygoparapophyseal lamina; prsdf, prezygapophyseal spinodiapophyseal fossa; prsl, prespinal lamina; prz, prezygapophysis; spdl, spinodiapophyseal lamina; spof, spinopostzygapophyseal fossa; sprl, spinoprezygapophyseal lamina; tpol, interpostzygapophyseal lamina; tprl, interprezygapophyseal lamina.

CPPLIP-036 and 110 (anterior trunk vertebrae, Fig. 6). These two vertebrae possess similar anatomy and preservation, both lacking the distal tips of the neural spines. The condyles are robust, expanding anteroposteriorly for one third the length of the respective centra. CPPLIP-110 possesses a rounded cotyle, whereas that of CPPLIP-036 is dorsoventrally expanded. The lateral and ventral surfaces of the centra are slightly concave anteroposteriorly. The pneumatic fossae are deep, reaching the medial portion of the centra and extending from the posterior portion of the condyles to the anterior margin of the cotyles.

On the anterior surfaces, the prezygapophyses extend anterodorsally with their articular facets facing mediodorsally. In CPPLIP-110, they surpass the posterior margin of the condyle, whereas those of CPPLIP-036 are positioned immediately above it. The prezygapophyses are posteromedially connected to the anterior margins of the neural spines by the spinoprezygapophyseal lamina. In CPPLIP-036, the left prezygoparapophyseal lamina delimits a small centroprezygapophyseal fossa dorsally.

The spinoprezygapophyseal laminae of CPPLIP-110 extend subparallel to the prespinal lamina and are separated from it by the spinoprezygapophyseal fossae. The spinoprezygapophyseal laminae also delimit shallow prezygapophyseal spinodiapophyseal fossae medially. In CPPLIP-036, the spinoprezygapophyseal lamina is absent, so the prezygapophyseal spinodiapophyseal fossa is bound laterally by the spinodiapophyseal lamina. In both vertebrae, the spinodiapophyseal laminae connect the diapophyses dorsolaterally to the neural spines and delimit the postzygapophyseal spinodiapophyseal fossae anteriorly.

The neural spines are dorsoventrally short, with triangular outlines in anterior/posterior views. Along their posterolateral edges, the spinopostzygapophyseal laminae extend to the postzygapophyses. Those are wide with oval shaped articular facets that face ventrolaterally. The postzygapophyses are connected anteroventrally to the postspinal laminae in CPPLIP-110 and directly to the base of the neural spine in CPPLIP-036; both are limited ventrally by the dorsal portion of the centropostzygapophyseal fossa. The postzygapophyses are connected ventrally to the centra *via* the centropostzygapophyseal laminae. Those laminae limit laterally the centropostzygapophyseal fossa. In CPPLIP-110, as seen only below the right postzygapophyses, that fossa corresponds to a small perforation, whereas they are larger in CPPLIP-036, with almost half the cotyle height. The centropostzygapophyseal laminae also limit the postzygapophyseal centrodiapophyseal fossae medially.

On the lateral surfaces, the diapophyses are connected medioposteriorly to the neural spines by the spinodiapophyseal laminae. The parapophyses of CPPLIP-110 are placed immediately above the posterior margin of the condyle, whereas those of CPPLIP-036 delimit the parapophyseal centrodiapophyseal fossae anteriorly. In CPPLIP-110, the parapophyseal centrodiapophyseal fossa is deep and divided in anterior and posterior portions by a thin paradiapophyseal lamina. The parapophyseal centrodiapophyseal fossae are bordered posteriorly by the posterior centrodiapophyseal laminae, which lie on the posterodorsal margins of the pneumatic fossae, and anteriorly by the prezygoparapophyseal laminae, which lie on the ventral margins of the parapophyses. The latter possess large rounded articular facets, which border dorsally the parapophyseal centrodiapophyseal fossae.

CPPLIP-036 possesses a slightly different laminar pattern. The parapophyseal centrodiapophyseal fossa is larger, with the posterior portions limited anterodorsally by thin accessory laminae. The centroparapophyseal fossae are limited anterodorsally by short paradiapophyseal laminae, which connect the diapophyses to the parapophyses. The latter also possess large rounded articular facets, but are positioned much more dorsally than those of CPPLIP-110. The parapophyses are also connected to the centrum *via* the anterior centroparapophyseal laminae and posteriorly by the posterior centroparapophyseal laminae.

**Figure 7:**
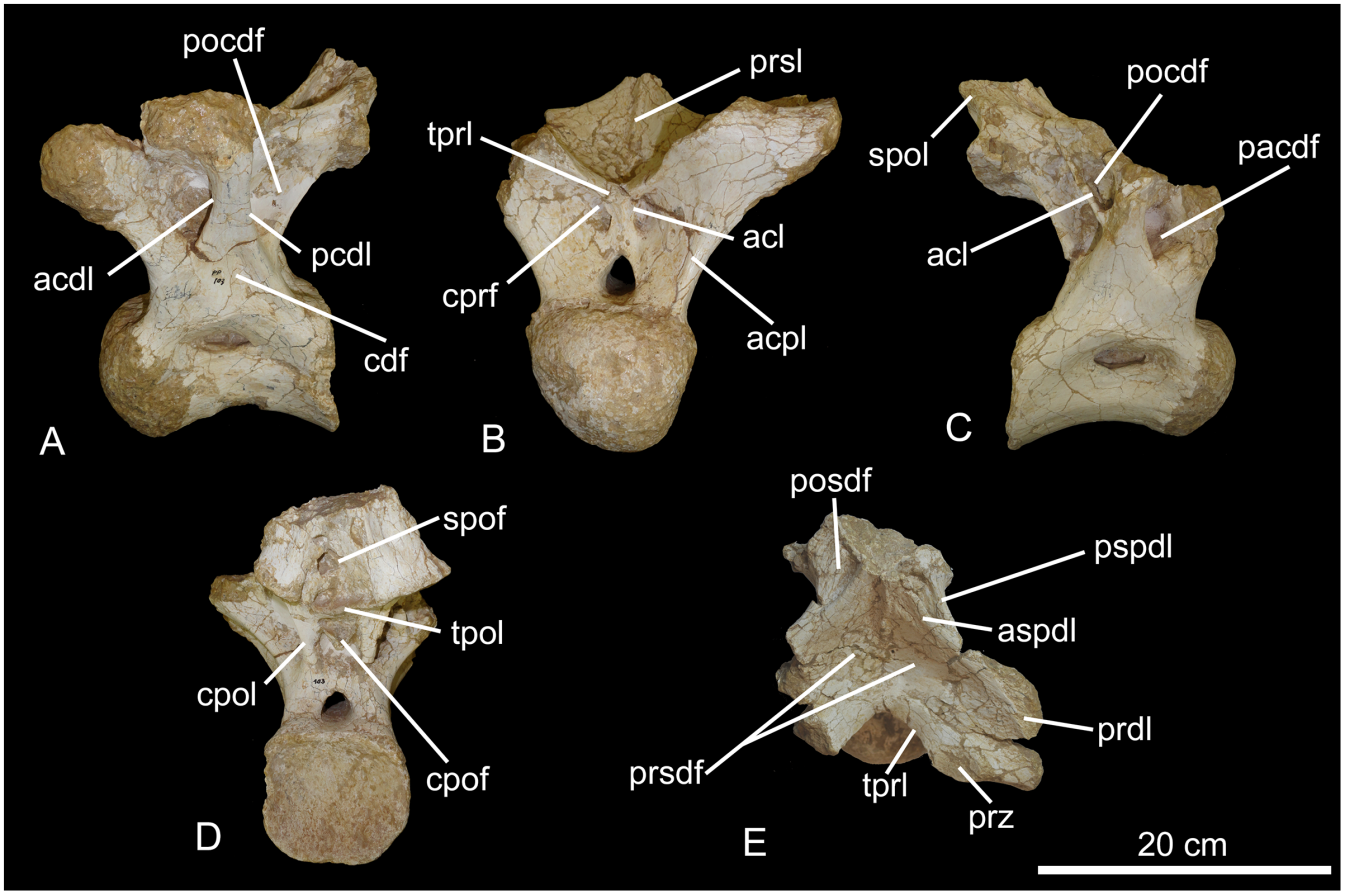
Middle trunk vertebrae of the BR-262 specimens. CPPLIP-103 in (A) left lateral; (B) anterior; (C) right lateral; (D) posterior and (E) dorsal views. Abbreviations: acl, accessory lamina; acpl, anterior centroparapophyseal lamina; aspdl, anterior ramus of the spinodiapophyseal lamina; cdf, centrodiapophyseal fossa; cpof, centropostzygapophyseal fossa; cpol, centropostzygapophyseal lamina; cprf, centroprezygapophyseal fossa; pacdf, parapophyseal centrodiapophyseal fossa; pcdl, posterior centrodiapophyseal lamina; pocdf, postzygapophyseal centrodiapophyseal fossa; posdf, postzygapophyseal spinodiapophyseal fossa; poz, postzygapophyses; prpl, prezygoparapophyseal lamina; prsdf, prezygapophyseal spinodiapophyseal fossa; pspdl, posterior ramus of the spinodiapophyseal lamina; prdl, prezygodiapophyseal lamina, prsl, prespinal lamina; spof, spinopostzygapophyseal fossa; sprl, spinoprezygapophyseal lamina; sprf, spinoprezygapophyseal fossa; tpol, interpostzygapophyseal lamina; tprl, interprezygapophyseal lamina.

**Figure 8:**
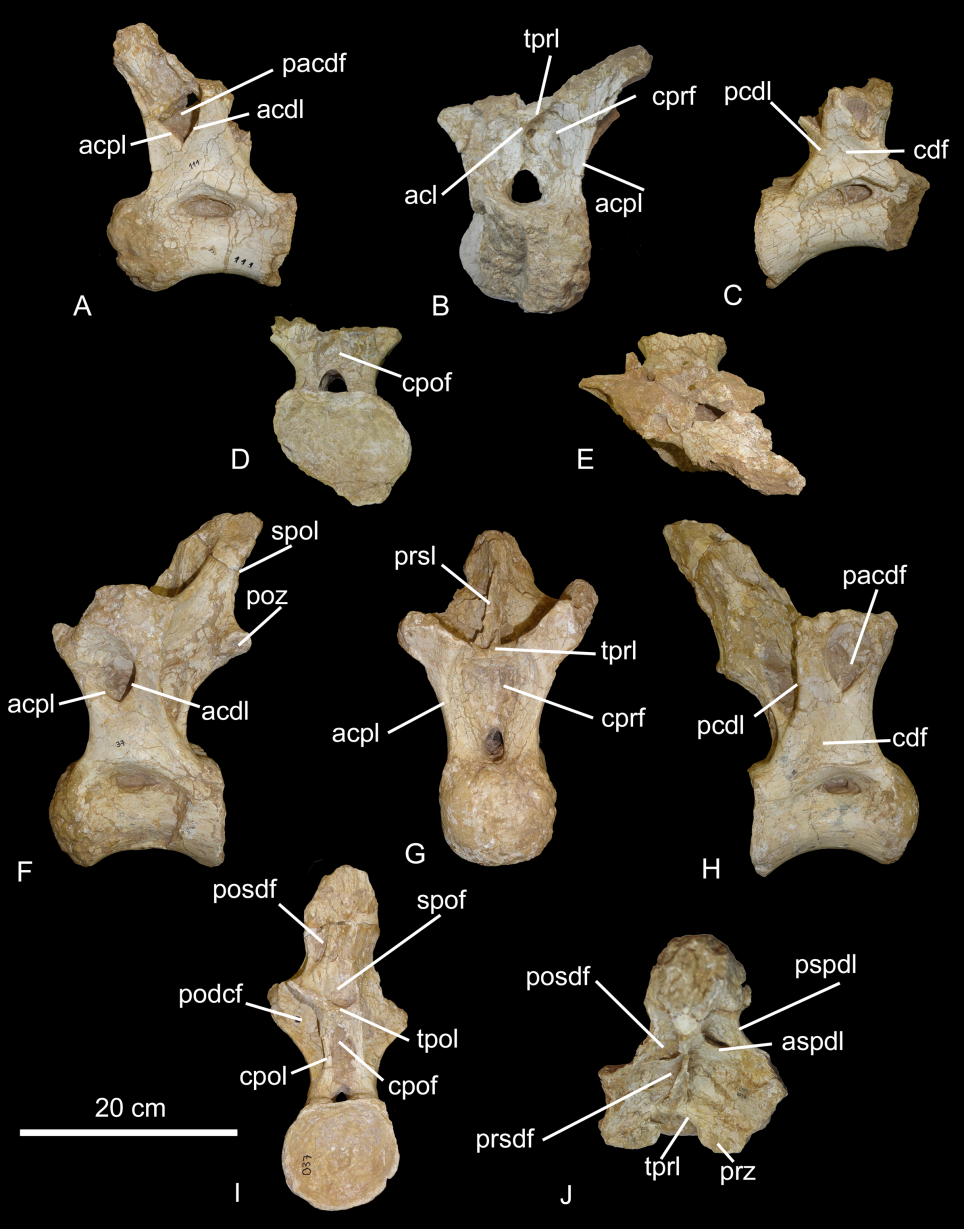
Middle trunk vertebrae of the BR-262 specimens. CPPLIP-111 in (A) left lateral; (B) anterior; (C) right lateral; (D) posterior and (E) dorsal views. CPPLIP-037 in (F) left lateral; (G) anterior; (H) right lateral; (I) posterior and (J) dorsal views. Abbreviations: acl, accessory lamina; acdl, anterior centrodiapophyseal lamina; acpl, anterior centroparapophyseal lamina; aspdl, anterior spinodiapophyseal lamina; cdf, centrodiapophyseal fossa; cpof, centropostzygapophyseal fossa; cpol, centropostzygapophyseal lamina; cprf, centroprezygapophyseal fossa; pacdf, parapophyseal centrodiapophyseal fossa; pcdl, posterior centrodiapophyseal lamina; pcpl, posterior centroparapophyseal lamina; pocdf, postzygapophyseal centrodiapophyseal fossa; pocdfl, postzygapophyseal centrodiapophyseal fossa lamina; posdf, postzygapophyseal spinodiapophyseal fossa; poz, postzygapophyses; prsdf, prezygapophyseal spinodiapophyseal fossa; pspdl, posterior spinodiapophyseal lamina; spof, spinopostzygapophyseal fossa; spol, spinopostzygapophyseal lamina; sprl, spinoprezygapophyseal lamina; tpol, interpostzygapophyseal lamina; tprl, interprezygapophyseal lamina.

CPPLIP-103 and CPPLIP-111 (middle trunk vertebrae, Figs. 7 and 8A–8E). CPPLIP-103 lacks the apex of the neural spine and the left parapophysis and diapophysis, whereas CPPLIP-111 preserves only the centrum, the most proximal portion of the neural arch, and the left parapophysis.

The condyles are robust and dorsoventrally expanded. The cotyle of CPPLIP-103 has a rounded shape, whereas that of CPPLIP-111 is dorsoventrally expanded. The lateral surfaces of the centra are more concave anteroposteriorly than those of the most anterior trunk vertebrae, whereas the ventral surfaces are also slightly anteroposteriorly concave in lateral view. The pneumatic fossae are deep and pierced by pneumatic foramina, extending from the posterior portion of the condyles to posterior centrodiapophyseal laminae. These foramina are inserted in concavities and the right pneumatic fossa of CPPLIP-111 is divided in anterior and posterior portions by a thin vertical lamina.

On the anterior surface, the prezygapophyses extend anteriorly, with their articular facets in CPPLIP-103 positioned immediately above the condyle, facing dorsomedially. The prezygapophyses are connected to the anterior margin of the neural spines posteromedially by the spinoprezygapophyseal lamina. On both vertebrae, small centroprezygapophyseal fossae are visible, delimited medially by an accessory vertical lamina and dorsally by the interprezygapophyseal lamina.

On the lateral surfaces, the diapophyses are connected posterodorsally to the postzygapophyses *via* the postzygodiapophyseal laminae. The diapophyses are connected to the centra anteroventrally by the anterior centrodiapophyseal laminae and posteroventrally by the posterior centrodiapophyseal laminae. The anterior centrodiapophyseal laminae posteriorly delimit deep parapophyseal centrodiapophyseal fossae, which are bordered anteriorly by the anterior centroparapophyseal laminae.

The spinodiapophyseal laminae present on CPPLIP-103 are divided into an anterior and a posterior portion, extending laterally from the apex of the neural spine and delimiting a shallow fossa between them. Both anterior and posterior portions of the spinodiapophyseal laminae connect the neural spines to the diapophyses and are separated by shallow postzygapophyseal spinodiapophyseal fossae. In lateral view, the neural spine of CPPLIP-103 angles posterodorsally, surpassing the cotyle. The spinopostzygapophyseal laminae limit the neural spines posteriorly, and extend to the postzygapophyses, which are wide, oval in shape, and their articular facets face ventrolaterally. The postzygapophyses are limited medially by deep spinopostzygapophyseal fossae and connected ventrally to the centra *via* centropostzygapophyseal laminae. Those laminae delimit deep centropostzygapophyseal fossae laterally and the postzygapophyseal centrodiapophyseal fossae anteriorly.

CPPLIP-037 (middle trunk vertebra, Figs. 8F–8J). This vertebra lacks the apex of the neural spine and both parapophyses and diapophyses. The condyle is short and do not surpass the prezygapophyses anteriorly. The cotyle is subcircular in posterior view and extends posteriorly beyond the postzygapophyses. The pneumatic fossae are deep and located on the dorsal margin of the centrum. On the anterior surface, the prezygapophyses extend anteromedially. Their facets face dorsomedially and are mediolaterally expanded. The prezygapophyses are connected to the anterior margin of the neural spine *via* the interprezygapophyseal lamina. This lamina delimits dorsally the deep centroprezygapophyseal fossa. In lateral view, the neural spine leans posterodorsally, reaching the posterior margin of the cotyle. The neural spine is limited posteriorly by the spinopostzygapophyseal laminae, which extend subparallel to the posterior ramus of the spinodiapophyseal laminae, creating small postzygapophyseal spinodiapophyseal fossae, only visible on the left side. Both laminae reach the postzygapophyses dorsally.

Only the left postzygapophysis is preserved. It has an oval shape and its facet faces ventrolaterally. It would be connected to the other postzygapophysis by the interpostzygapophyseal lamina, which also delimits ventrally the spinopostzygapophyseal fossa. The postzygapophysis is connected ventrally to the centrum by the centropostzygapophyseal lamina. This lamina limits the centropostzygapophyseal fossa laterally and posteromedially the postzygapophyseal centrodiapophyseal fossa. On the lateral surfaces, the diapophyses are connected ventrolaterally to the centrum by the posterior centrodiapophyseal laminae, which extend to the posterior margin of the centrum. The diapophyses limit dorsally the parapophyseal centrodiapophyseal fossa, which are also limited anteriorly by the anterior centroparapophyseal laminae and posteriorly by the anterior centrodiapophyseal lamina.

**Figure 9:**
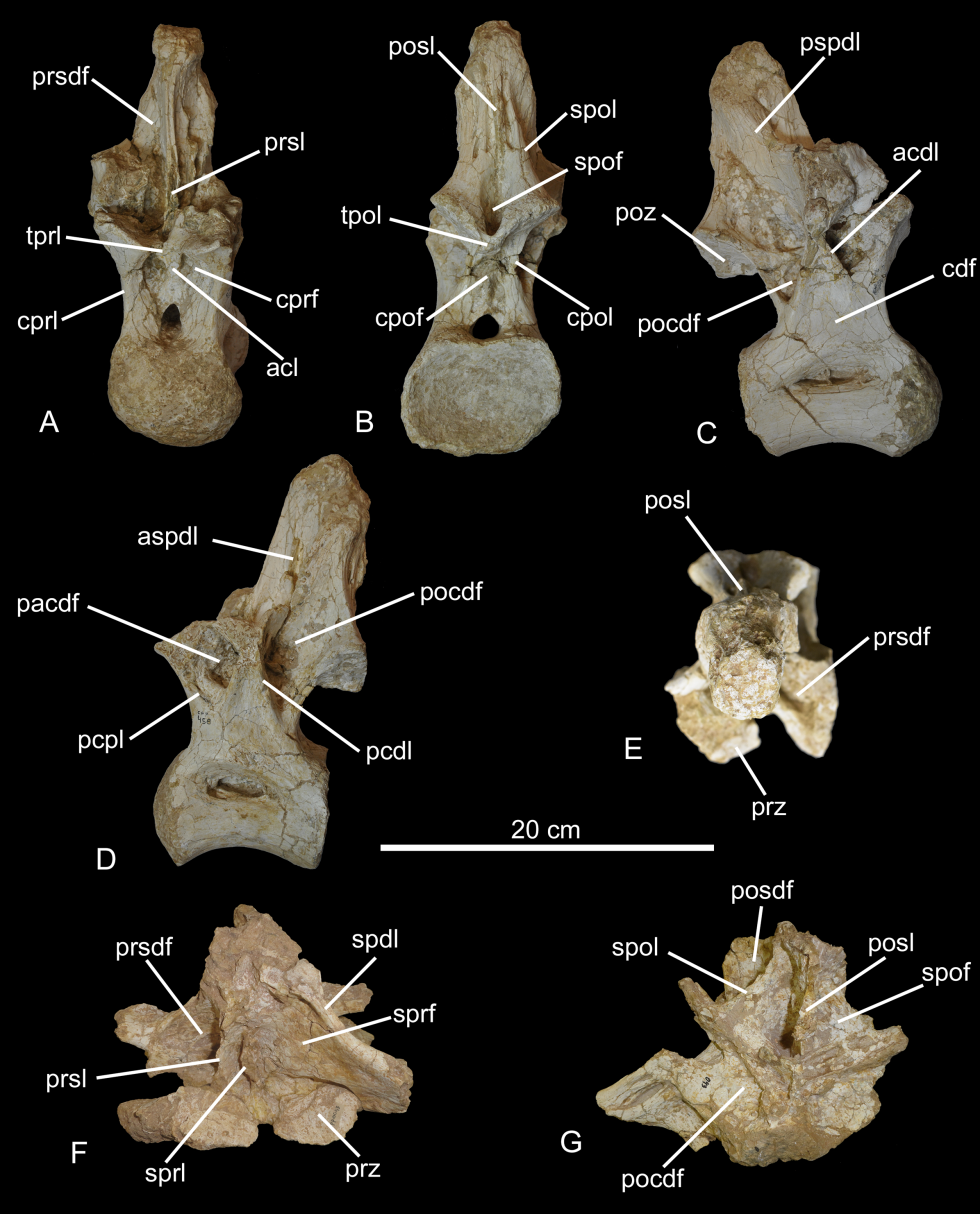
Posterior trunk vertebrae of the BR-262 specimens. CPPLIP-458 in (A) anterior; (B) posterior; (C) right lateral; (D) left lateral and (E) dorsal views. CPPLIP-043 in (F) dorsal and (G) posteroventral views. Abbreviations: acl, accessory lamina; acdl, anterior centrodiapophyseal lamina; aspdl, anterior spinodiapophyseal lamina; cdf, centrodiapophyseal fossa; cpof, centropostzygapophyseal fossa; cpol, centropostzygapophyseal lamina; cprf, centroprezygapophyseal fossa; pacdf, parapophyseal centrodiapophyseal fossa; pcdl, posterior centrodiapophyseal lamina; pcpl, posterior centroparapophyseal lamina; pocdf, postzygapophyseal centrodiapophyseal fossa; prsdf, prezygapophyseal spinodiapophyseal fossa; pspdl, posterior spinodiapophyseal lamina; prsl, prespinal lamina; prz, prezygapophysis; spdl, spinodiapophyseal lamina; spof, spinopostzygapophyseal fossa; spol, spinopostzygapophyseal lamina; sprl, spinoprezygapophyseal lamina; tpol, interpostzygapophyseal lamina; tprl, interprezygapophyseal lamina.

CPPLIP-458 (posterior trunk vertebra, Figs. 9A–9E). This vertebra is well-preserved, only lacking the diapophyses and parapophyses. The condyle projects anteriorly and is less convex than those of more anterior vertebrae. The cotyle is transversely expanded and its posterior margin lies below the postzygapophyses. The pneumatic fossae are located on the dorsal margin of the centrum. The left one is deeper than the right, with a small depression on its anterodorsal margin. On the anterior surface, only the right prezygapophysis is preserved; its articular facet faces dorsomedially and is mediolaterally expanded. The interprezygapophyseal laminae limits the centroprezygapophyseal fossae dorsally, which are separated in the center by a vertical accessory lamina. The prezygapophyses are connected ventrally to the centrum by the centroprezygapophyseal lamina, which also limit laterally the centroprezygapophyseal fossae.

The neural spine has a triangular shape in lateral view, with a ‘bulbous’ apex, *i.e*., it is expanded transversely. It is connected to the diapophyses by the spinodiapophyseal laminae, which are divided dorsally in anterior and posterior rami, both of which limit the spinodiapophyseal lamina fossae. Each anterior spinodiapophyseal lamina—assuming the presence of this lamina instead of a spinoprezygapophyseal lamina, as seen on the anterior elements—extends parallel to the robust prespinal lamina and is separated from it by the spinoprezygapophyseal fossa. The neural spine is connected posteroventrally to the postzygapophyses by spinopostzygapophyseal laminae. These laminae extend parallel to the postspinal lamina and are separated from it by the spinopostzygapophyseal fossae. The postzygapophyses are wide, with rounded facets that face ventrolaterally, and which are connected to one another by a short interpostzygapophyseal lamina—which also delimits the centropostzygapophyseal fossa dorsally—and to the centrum by the centropostzygapophyseal laminae. Such laminae also limit the postzygapophyseal centrodiapophyseal fossae posteriorly. On the lateral surface, the posterior centrodiapophyseal lamina extends posteroventrally from the diapophysis to the posterior margin of the neural arch, and limit the postzygapophyseal centrodiapophyseal fossa anteriorly. A small parapophyseal centrodiapophyseal fossa is visible in lateral view, which is limited anteriorly by the posterior centroparapophyseal lamina and posteriorly by the centrodiapophyseal lamina.

CPPLIP-043 (posterior trunk neural arch fragment, Fig. 9). The prezygapophyses are displaced laterally, with wide articular facets facing dorsally, and connected to one another by a short interprezygapophyseal lamina. The spinodiapophyseal laminae extend laterally from the neural spine to the diapophyses. They limit a deep spinoprezygapophyseal fossa anteriorly, which is only present on the right side of the neural arch and limited medially by the spinoprezygapophyseal lamina. The latter also limits laterally a shallow prezygapophyseal spinodiapophyseal fossae anteriorly, which are divided in half by robust prespinal laminae. The neural spine is connected posterolaterally to the postzygapophyses by the spinopostzygapophyseal laminae, which also limit the postzygapophyseal centrodiapophyseal fossae posteriorly. The postzygapophyses are poorly preserved, lacking the articular facets. They limit the spinopostzygapophyseal fossae ventrally, which is separated on two portions by the postspinal lamina. The postzygapophyses also limit mediodorsally the postzygapophyseal centrodiapophyseal fossa.

*Trunk ribs*. Three isolated sauropod trunk rib fragments (Fig. 5) have been recovered from BR-262 locality: CPLIP-044, 097, and 108. The first two are distal fragments, composed mainly of a thin and flattened, laminar bone. CPPLIP-108 represents a proximal portion, with a shallow longitudinal groove on its anterior face.

*Caudal vertebrae*. Ten sauropod caudal vertebrae (CPPLIP-045, 046, 047, 061, 091, 093, 094, 095, 096, 102) were recovered from BR-262. Based on comparisons with more complete caudal series such as those of *Baurutitan britoi* (Kellner, Campos & Trotta, 2005), *Dreadnoughtus schrani* (Lacovara et al., 2014), and *Rapetosaurus krausei* (Curry Rogers, 2009), we identified the elements as one anterior, four middle, and five posterior caudal vertebrae.

**Figure 10:**
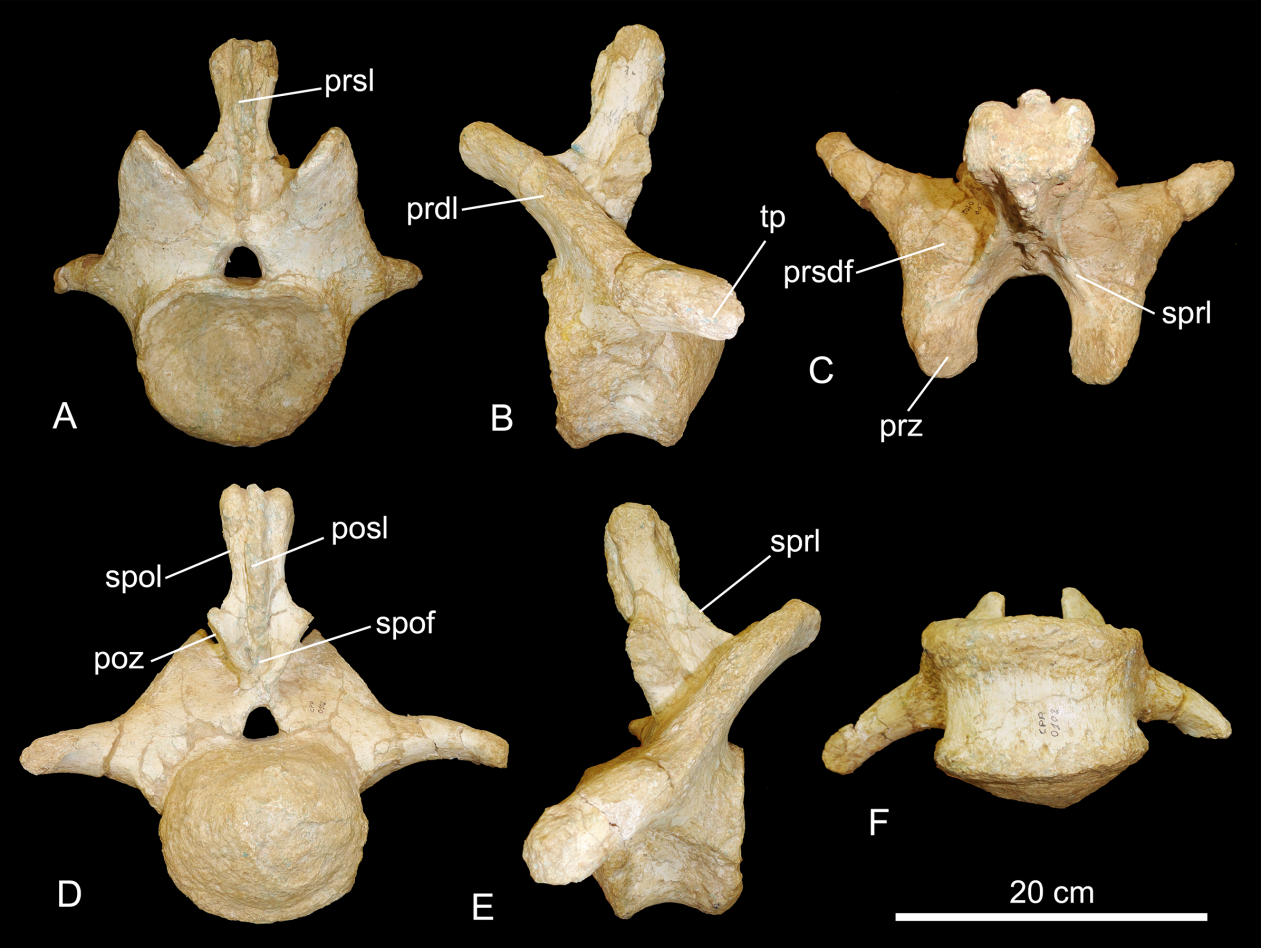
Most anterior caudal vertebra of the BR-262 specimens. CPPLIP-102 in (A) anterior; (B) left lateral; (C) dorsal; (D) posterior; (E) right lateral and (F) ventral views. Abbreviations: posl, postspinal lamina; poz, postzygapophyses; prdl, prezygodiapophyseal lamina; prsdf, prezygapophyseal spinodiapophyseal fossa; prsl, prespinal lamina; prz, prezygapophyses; spof, spinopostzygapophyseal fossa; spol, spinopostzygapophyseal lamina; sprl, spinoprezygapophyseal lamina; tp, transverse process.

CPPLIP-102 (anterior caudal vertebra, Fig. 10). The lateral and ventral surfaces of the centrum are slightly anteroposteriorly concave. The centrum has an aEI of 0.7. The condyle is strongly convex, corresponding to almost half of the remaining length of the centrum. The cotyle is shallow and with a sub-oval outline. The neural spine is transversely expanded in its distal half, creating an ellipse-like format in dorsal view, and leans gently posteriorly. It is connected to the prezygapophyses by short spinoprezygapophyseal laminae. Such laminae extend parallel to a robust prespinal lamina and are separated from it by a shallow spinoprezygapophyseal fossa. The prezygapophyses project anteriorly and are connected to the transverse processes *via* the prezygodiapophyseal laminae. The transverse processes are laterally projected, with their most distal portions leaning posteriorly, surpassing the posterior margin of the condyle. On the posterior surface, the postzygapophyses are connected to the neural spine by the spinopostzygapophyseal laminae, which have their most distal portions mediolaterally expanded, creating a ‘bulbous’ outline in posterior view. Such laminae extend parallel to a robust postspinal lamina, which contacts ventrally a small interpostzygapophyseal lamina. The postzygapophyses are wide, with articular facets that are dorsoventrally expanded and face ventrolaterally.

**Figure 11:**
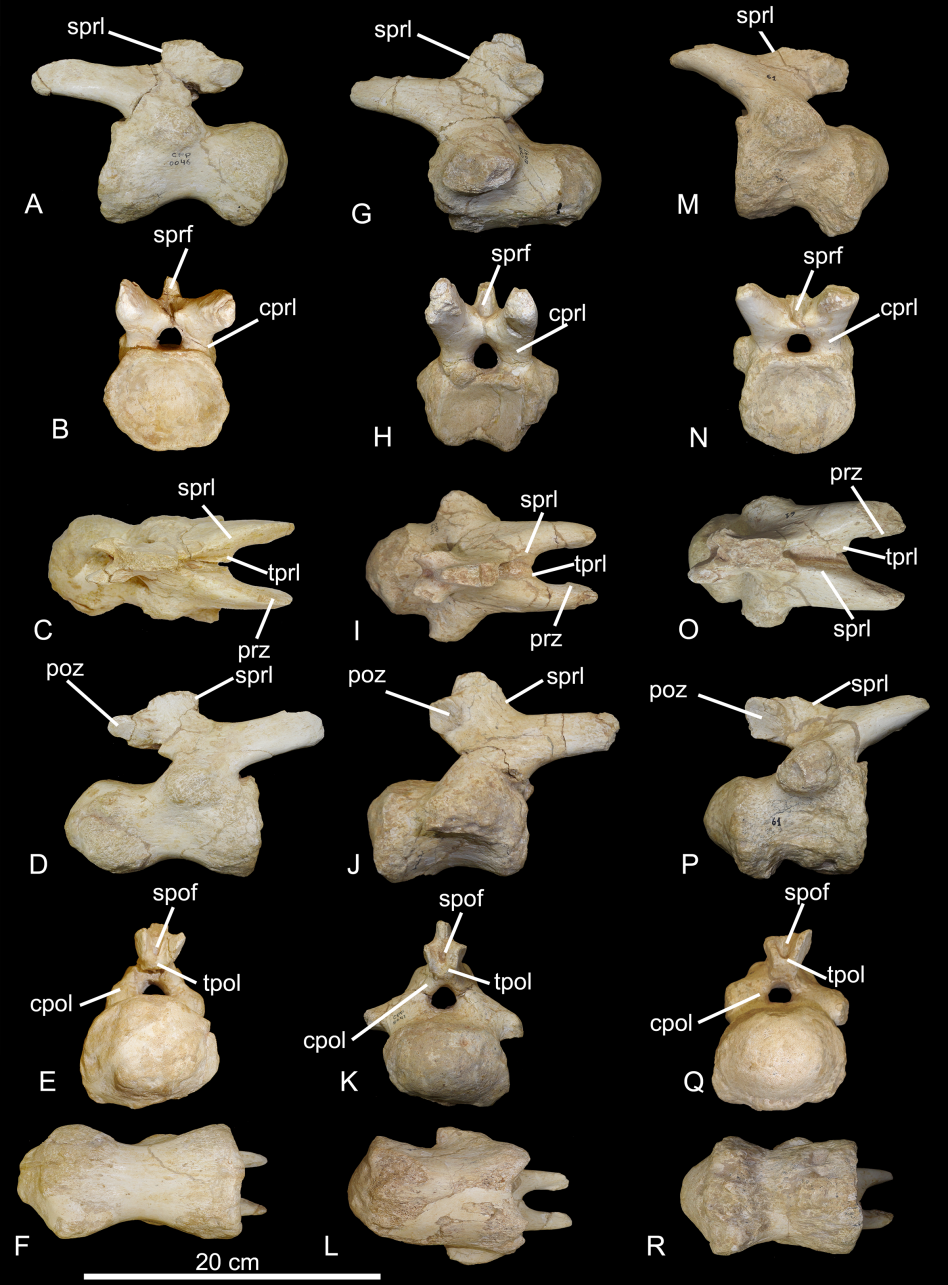
Middle caudal vertebrae of the BR-262 specimens. CPPLIP-046 in (A) left lateral; (D) anterior; (G) dorsal; (J) right lateral; (M) posterior and (P) ventral views. CPPLIP-047 in (B) left lateral; (E) anterior; (H) dorsal; (K) right lateral; (N) posterior and (Q) ventral views. CPPLIP-061 in (C) left lateral; (F) anterior; (I) dorsal; (L) right lateral; (O) posterior and (R) ventral views. Abbreviations: cpol, centropostzygapophyseal lamina; cprl, centroprezygapophyseal lamina; poz, postzygapophyses; prz, prezygapophyses; spol, spinopostzygapophyseal lamina; sprf, spinoprezygapophyseal fossa; sprl, spinoprezygapophyseal lamina; tprl, interprezygapophyseal; tpol, interpostzygapophyseal lamina.

CPPLIP-046, 047, and 061 (middle caudal vertebrae, Fig. 11). These vertebrae possess a similar anatomy. All structures are preserved in CPPLIP-047 and 061, except the distalmost portion of the neural spines and the most distal portions of the transverse processes, whereas only the proximal portions of the transverse processes are preserved in CPPLIP-046. CPPLIP-046 and 047 have their lateral and ventral surfaces slightly concave anteroposteriorly. CPPLIP-061 has slightly anteroposteriorly concave lateral surfaces, whereas its ventral surface is strongly anteroposteriorly concave, with the condyle dorsoventrally taller than the cotyle. Posterior chevron facets are visible on both CPPLIP-046 and CPPLIP-061, but were not preserved on CPPLIP-047. They project ventrolaterally from the distal portion of the condyles and have triangular shapes in dorsal view.

The centra possess an aEI of 1.2 (CPPLIP-046), 1.1 (CPPLIP-047) and 0.9 (CPPLIP-061). The condyles are robust, projecting posterior to the postzygapophyses. That of CPPLIP-047 is dorsoventrally compressed, whereas those of CPPLIP-046 and 061 have rounded outlines. The cotyle of CPPLIP-046 is transversely compressed, whereas those of CPPLIP-047 and 061 have rounded outlines, all with well-defined margins. The neural spines are connected to the pre- and postzygapophyses *via* the spinoprezygapophyseal and spinopostzygapophyseal laminae, respectively. The transverse processes are poorly preserved and located anteriorly, near the cotyles. That of CPPLIP-047 is more robust, *i.e*., expanded dorsoventrally and projecting posteriorly.

The prezygapophyses are long (almost half the respective centrum length) and dorsoventrally flattened, their articular facets facing medially. They are connected to their counterparts by thin interprezygapophyseal laminae and to the neural spines by the spinoprezygapophyseal laminae, which extend until the apex of the neural spines, where they limit shallow spinoprezygapophyseal fossae. The prezygapophyses are posteriorly connected to the centra *via* centroprezygapophyseal laminae, which extend until the dorsal margins of the cotyles. The postzygapophyses are short, separated by thin interpostzygapophyseal laminae, with wide articular facets facing laterally. They are connected to the neural spines by the spinopostzygapophyseal laminae, which laterally delimit shallow spinopostzygapophyseal fossae. The postzygapophyses are connected to the centra—ventrally in CPPLIP-047 and anteroventrally in CPPLIP-046 and 061—*via* the centropostzygapophyseal laminae, which extend until the dorsal margin of the neural canals.

**Figure 12:**
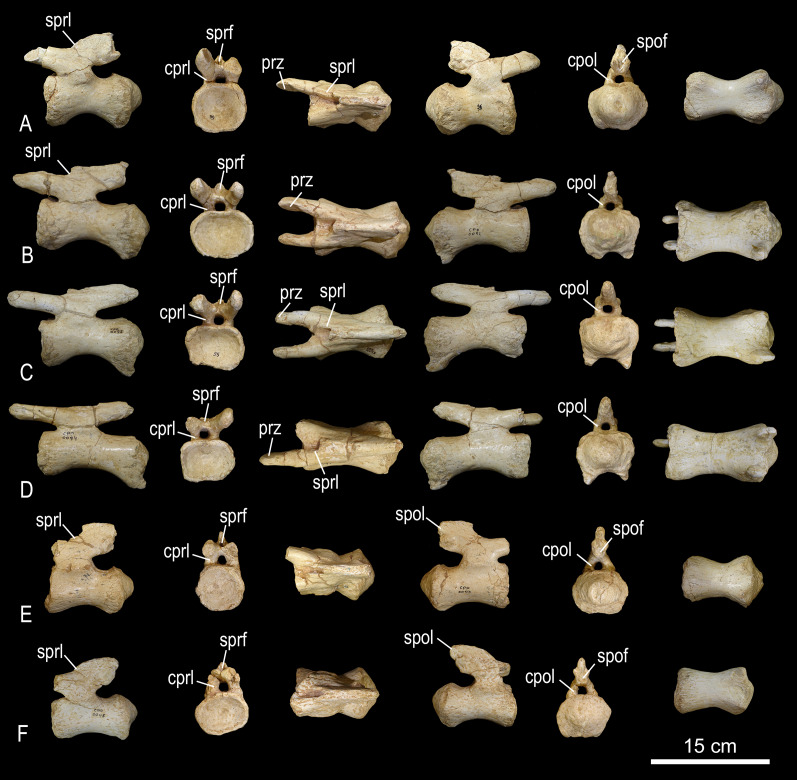
Middle and posterior caudal vertebrae of the BR-262 specimens. (A) CPPLIP-096 in left lateral; anterior; dorsal; right lateral; posterior and ventral views. (B) CPPLIP-091 in left lateral; anterior; dorsal; right lateral; posterior and ventral views. (C) CPPLIP-095 in left lateral; anterior; dorsal; right lateral; posterior and ventral views. (D) CPPLIP-094 in left lateral; anterior; dorsal; right lateral; posterior and ventral views. (E) CPPLIP-093 in left lateral; anterior; dorsal; right lateral; posterior and ventral views. (F) CPPLIP-045 in left lateral; anterior; dorsal; right lateral; posterior and ventral views. Abbreviations: cpol, centropostzygapophyseal lamina; cprl, centroprezygapophyseal lamina; spof, spinopostzygapophyseal fossa; spol, spinopostzygapophyseal lamina; sprf, spinoprezygapophyseal fossa; sprl, spinoprezygapophyseal lamina.

CPPLIP-096 (middle caudal vertebra, Fig. 12A). This vertebra lacks the distalmost portions of the neural spine and postzygapophyses. The ventral and lateral surfaces of the centrum are slightly concave anteroposteriorly, the former has four points for the chevron articulation, two below the condyle and two below the cotyle. The centrum has an aEI of 1.7. The condyle is strongly expanded anteroposteriorly, extends beyond the postzygapophyses and has a small slit extending ventrodorsally. The cotyle is shallow, with a rounded outline and well-defined margins. The neural spine is lateromedially narrow and connected to the pre- and postzygapophyses *via* the spinoprezygapophyseal and spinopostzygapophyseal laminae, respectively. Due to its more posterior position along the tail, the vertebra has transverse processes composed only by small lateral projections.

The prezygapophyses are long with the articular facets facing medially. They are connected posteriorly to the neural spine by the spinoprezygapophyseal laminae, which laterally limit shallow spinoprezygapophyseal fossae. The prezygapophyses are connected posteriorly to the centrum by the centroprezygapophyseal laminae, which extend anteriorly towards the cotyle. The postzygapophyses are connected to the neural spine by the spinopostzygapophyseal laminae, which laterally delimit shallow spinopostzygapophyseal fossae. They are connected to the centrum by the centropostzygapophyseal laminae, which extend until the dorsal margin of the neural canal.

CPPLIP-091, CPPLIP-094, and CPPLIP-095 (posterior caudal vertebrae, Fig. 12). These vertebrae are quite similar, with all structures preserved, except for the neural spine and the right prezygapophysis of CPPLIP-094. Their centra have convex lateral and ventral surfaces. CPPLIP-094 and 095 bear two processes below their condyles, which are remains of fused chevrons. The condyles extend posteriorly and are surrounded laterally by concave margins. The cotyles are deep, with rounded outlines and well-defined margins. Only the most proximal portion of the neural spine is preserved in CPPLIP-091. It is laterally narrow and connected to the pre- and postzygapophyses by the spinoprezygapo- and spinopostzygapophyseal laminae, respectively. The centra possess aEIs of 1.8 (CPPLIP-091), 1.9 (CPPLIP-094) and 1.6 (CPPLIP-095).

The prezygapophyses are long, with convex lateral margins. Their articular facets, only preserved on the right side of CPPLIP-091, are anteroposteriorly expanded and face medially. The spinoprezygapophyseal laminae laterally delimit shallow spinoprezygapophyseal fossae. The prezygapophyses are posteroventrally connected to the centra by the centroprezygapophyseal laminae, which extend until the lateral margins of the neural canals. The postzygapophyses are short, lack well preserved articular facets, and are connected posteroventrally to the centra by the centropostzygapophyseal laminae.

CPPLIP-093 and 045 (posterior caudal vertebrae, Fig. 12). These two vertebrae are the only articulated elements found at ‘Rodovia’ site. Their lateral and ventral surfaces are anteroposteriorly concave. The latter have two points for the articulation of the chevrons, below the condyles. The centrum aEI is 1.5 for CPPLIP-045 and 1.6 for CPPLIP-045. The condyles are slightly projected posteriorly and are surrounded by concave margins. CPPLIP-093 possess a small depression on the center of the condyle, whereas CPPLIP-045 has a small slit projecting dorsoventrally. The cotyles are shallow with rounded outlines. Only the most proximal portion of the neural spines are preserved. They are transversely narrow and connected to the prezygapophyses by the spinoprezygapophyseal laminae, which limit laterally shallow spinoprezygapophyseal fossae. The prezygapophyses are posteroventrally connected to the centra by the centroprezygapophyseal laminae, which extend until the dorsal margin of the neural canals. The postzygapophyses are short, with rounded articular facets that face laterally, and also form the lateral limits of shallow spinopostzygapophyseal fossae. They are anteroventrally connected to the centra by short centropostzygapophyseal laminae, which extend until the dorsal margin of the neural canals.

*Chevrons*. Nine sauropod chevrons were recovered from the BR-262 site, seven from the anterior and two from the posterior portions of the tail.

**Figure 13:**
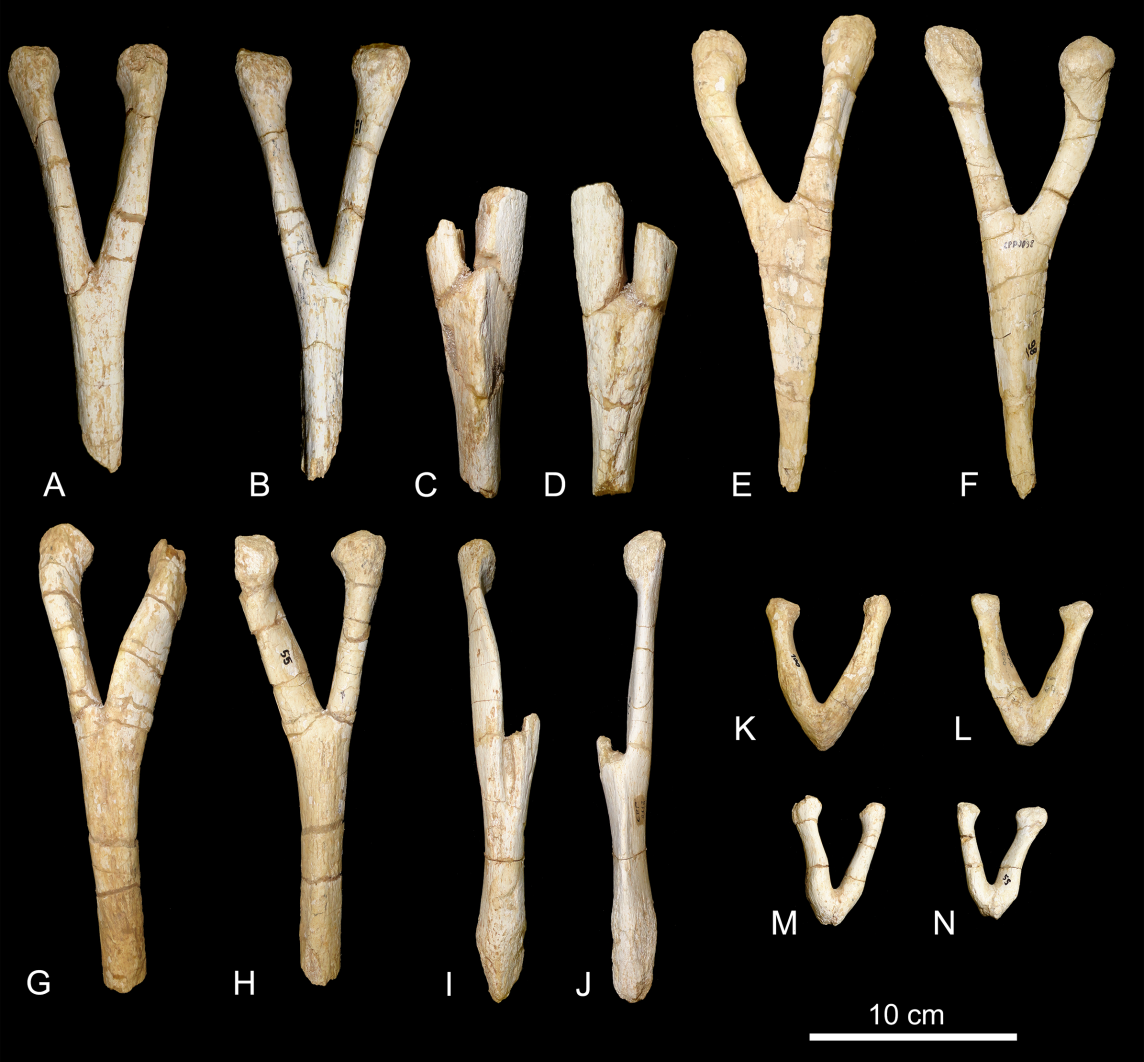
Anterior and posterior chevrons of the BR-262 specimens. CPPLIP-099 (anterior chevron) in (A) anterior and (B) posterior views. CPPLIP-056 (anterior chevron) in (C) anterior and (D) posterior views. CPPLIP-098 (anterior chevron) in (E) anterior and (F) posterior views. CPPLIP-055 (anterior chevron) in (G) anterior and (H) posterior views. CPPLIP-112 (anterior chevron) in (I) anterior and (J) posterior views. CPPLIP-100 (posterior chevron) in (K) anterior and (L) posterior views. CPPLIP-055 (posterior chevron) in (M) anterior and (N) posterior views.

CPPLIP-055, 056, 098, 099, 112, and 188 (anterior chevrons, Fig. 13). The haemal canals are dorsally open. The articular facets are composed of single surfaces, without divisions, and those from CPPLIP-055 and 098 are posteriorly inclined. The preserved distal rami of the chevrons represent almost two thirds of their total length. They are transversely flattened and some of the elements possess an anteriorly projected crest (CPPLIP-056 and CPPLIP-059), whereas the others bear a small depression (CPPLIP-055, CPPLIP-098, CPPLIP-099 and CPPLIP-112). On their posterior surfaces all elements possess a posteriorly projected crest.

CPPLIP-057 and 100 (posterior chevrons, Fig. 13). Only their proximal rami are preserved. Each of the elements has a small crest projecting anterolaterally and bear a dorsally open haemal canal. The articular facets are poorly preserved, but are undivided.

### Appendicular skeleton

Titanosaur appendicular remains recovered from BR-262 site include: right pectoral girdle (scapula, coracoid) and sternal plate, right and left humeri, possible right metacarpal I, right and left ischia, and possible left metatarsals III and IV.

**Figure 14:**
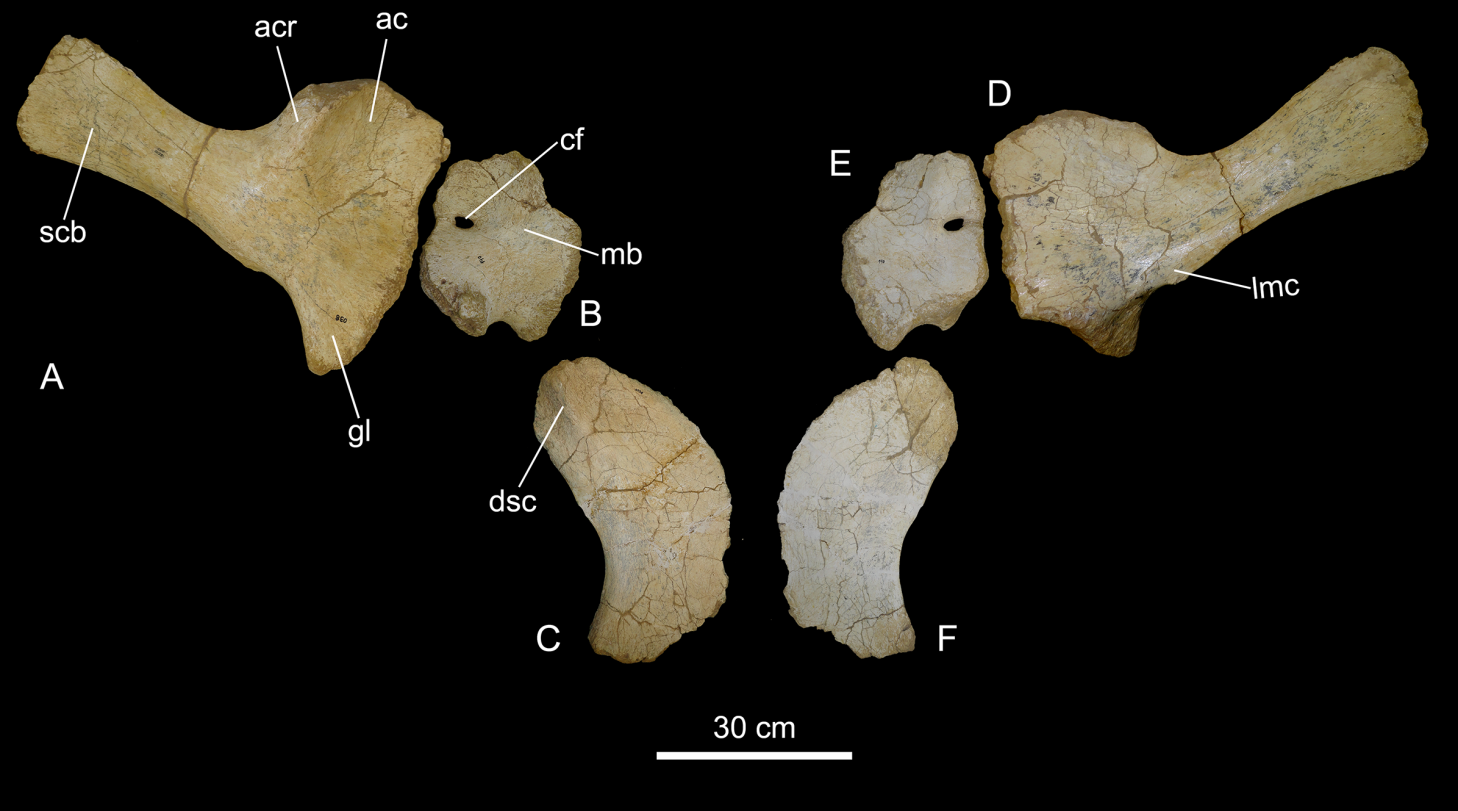
Scapular girdle and sternal plate of the BR-262 specimens. CPPLIP-038 (right scapula) in (A) lateral and (D) medial views. CPPLIP-140 (right coracoid) in (B) lateral and (E) medial views. CPPLIP-138 (right sternal plate) in (C) ventral and (F) dorsal views. Abbreviations: ac, acromion; acr, acromial ridge; cf, coracoid foramen; dsc, dorsoventrally projected crest; lmc, lateromedially projected crest; mb, medial bulge; scb, scapular blade.

*Pectoral girdle*. CPPLIP-038 (right scapula, Fig. 14). The scapula is described here with the long axis of the blade oriented horizontally and its external surface facing laterally. The lateral surface of the acromion plate is slightly anteroposteriorly concave and limited posteriorly by a robust acromial ridge, which represents the insertion of *M. deltoideus clavicularis*. The scapular glenoid is laterally deflected and expands ventrally, with a subtriangular outline when seen in lateral/medial view. Its ventralmost portion acts as the insertion for *M. triceps*. The glenoid medially bounds a small mediolaterally oriented crest, which is the insertion for *M. scapulohumeralis posterior*. The scapular blade extends posteriorly as a flat lamina, with a subrectangular cross section and a subsquared posterior end. It has a small ridge on the lateral surface where *M. serratus superficialis* was inserted. On its dorsal surface, the scapula is limited laterally and medially by a pair of anteroposteriorly extending crests.

CPPLIP-140 (right coracoid, Fig. 14). The bone is poorly preserved and has a rounded outline when seen in medial/lateral view. Although not complete, the dorsal margin of the coracoid is at about the same level as that of the scapula, with a small medial projection. The medial face is slightly concave on its more proximal portion. The glenoid fossa is well preserved and strongly excavated with a mediolaterally-expanded lateral margin. Anterior to that, a marked bulge represents the insertion of *M. coracobrachialis brevis*. The coracoid foramen is located on the posterior portion of the bone, near the scapular articulation. Anteroventral to the coracoid foramen, a convex surface acts as the insertion for the *M. biceps*.

CPPLIP-138 (right sternal plate, Fig. 14). The sternal plate is a flat, laminar bone, expanded lateromedially on both anterior and posterior ends, creating the typical kidney-shape common in titanosaurs (Salgado, Coria & Calvo, 1997). The medial margin is convex, whereas the lateral is concave. Its ventral surface bears a small anteroposteriorly oriented crest that bounds a lateral concavity.

**Figure 15:**
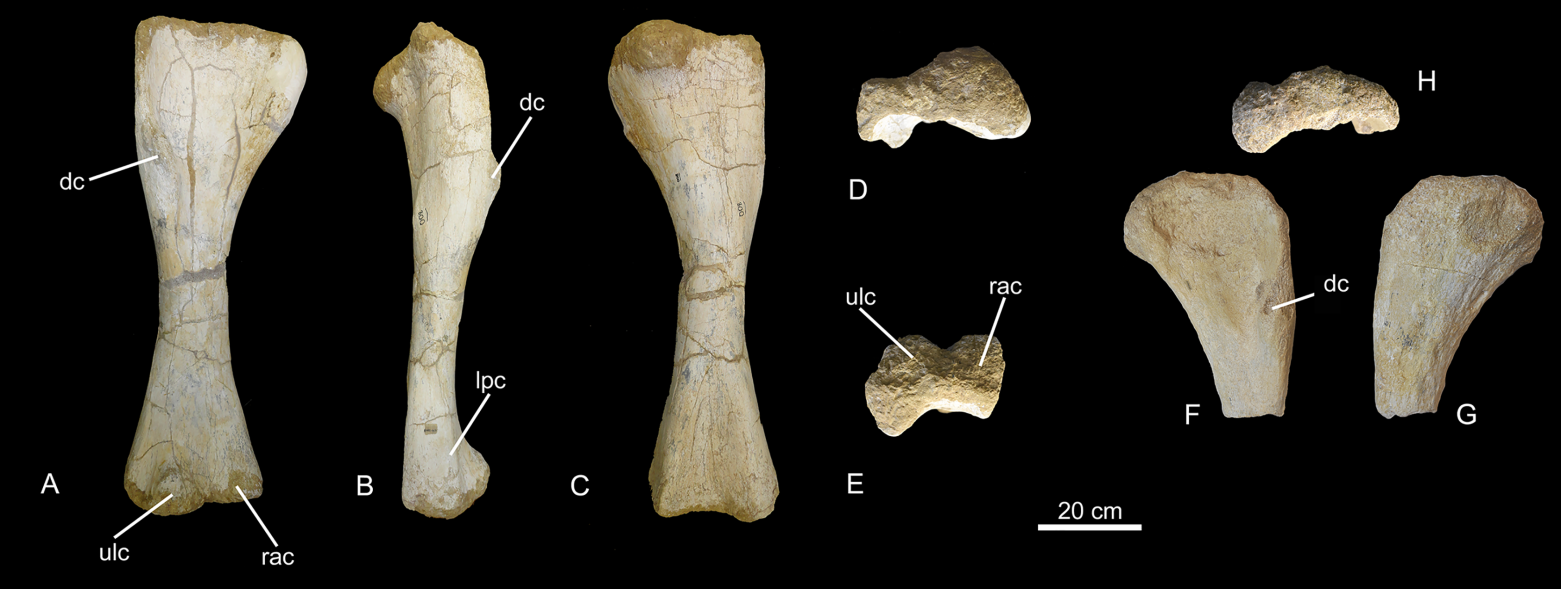
Humeri of the BR-262 specimens. CPPLIP-008 (right humerus) in (A) anterior; (B) lateral; (C) posterior; (D) proximal and (E) distal views. CPPLIP-007 (left humerus) in (F) anterior; (G) posterior and (H) proximal views. Abbreviations: dc, deltapectoral crest; lpc, laterally projected crest; rac, radial condyle; ulc, ulnar condyle.

*Forelimb* (Fig. 15). CPPLIP-008 (right humerus) and 007 (proximal portion of left humerus) are likely paired, whereas CPPLIP-263 (proximal portion of left humerus) is a much larger element. Because it cannot be assigned to the same specimen as the other BR-262 remains, it is not described here. The humeri are gracile elements (ECC (eccentricity index) for CPPLIP-008: 1.2), with similar anatomy that are described together, with the differences cited when necessary. The deltopectoral crest projects anteriorly from the lateral margin of the proximal portion of the bone and is slightly medially deflected. It extends distally until half the length of the bone, with its mediolateral thickness almost doubling towards its distal end. Its lateral surface marks the insertion for *M. scapular deltoid*, whereas its proximal margin received *M. pectoralis*. Proximally on the posterior surface of the humeral head, a concavity extends mediolaterally, representing the insertion of *M. coracobrachialis brevis*. The medial border of the head expands anteriorly, forming a bulge, which represents the insertion for *M. supracoracoideus*.

At mid-shaft, the humerus has a sub-circular cross-section, slightly compressed anteroposteriorly. In the distal portion, the radial and ulnar condyles are pronounced. The former is anteriorly expanded, limited both medially and laterally by shallow fossae, creating a triangular outline in anterior view. Its anterior surface is slightly concave, without divisions. The lateral fossa separates the ulnar condyle from a laterally projecting crest. The first represents the insertion of both *Mm. extensor carpi radialis* and *extensor digitalis communis*, whereas the last received *M. extensor carpi ulnaris*. The radial condyle is more robust, expanded both proximodistally and lateromedially. Its anterior surface represents the insertion for *M. corobrachialis longus*. On the posterior surface of the distal third of the bone there is a deep supracondylar fossa bound by both medial and lateral ridges.

**Figure 16:**
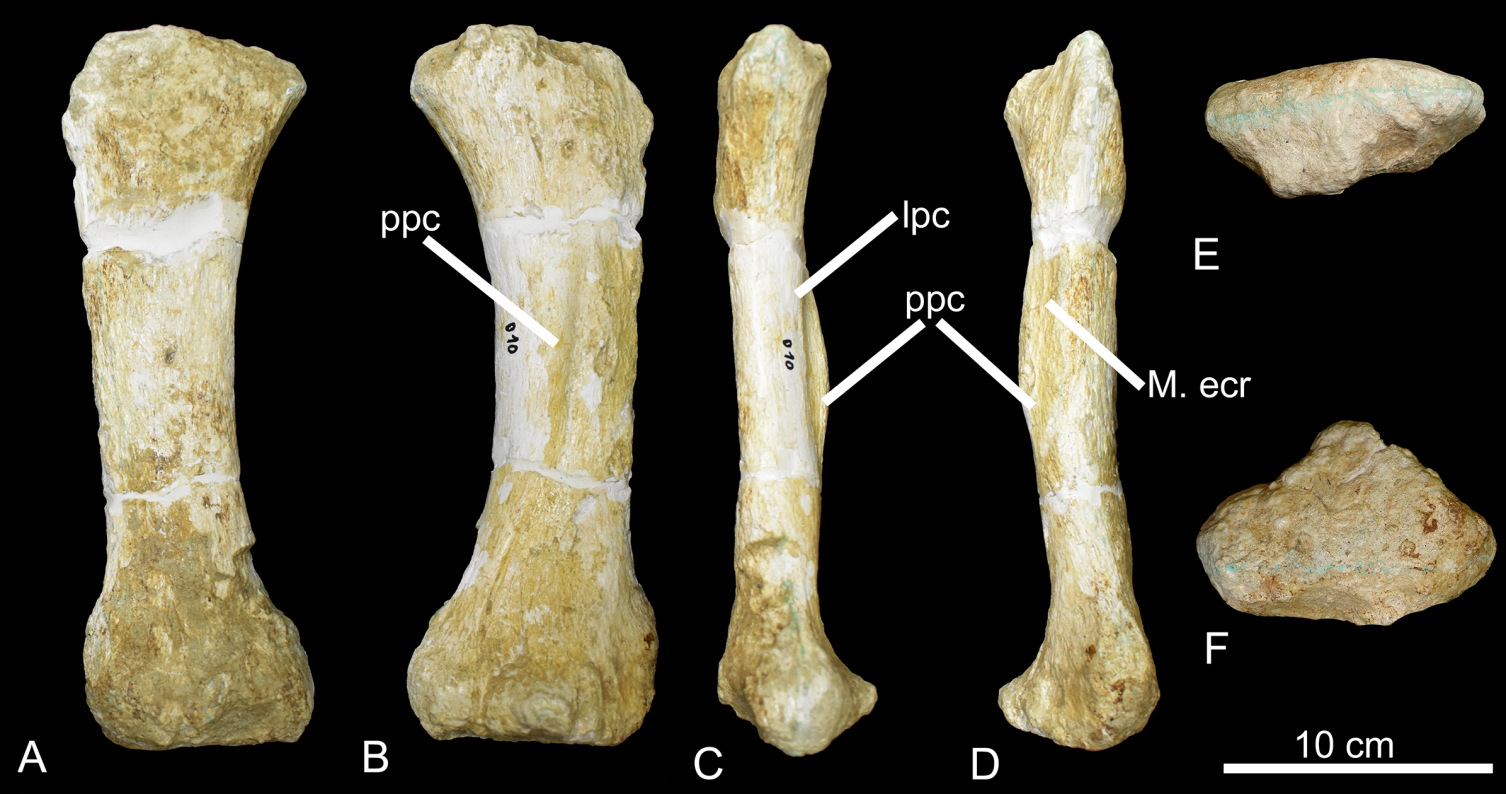
Metacarpal of the BR-262 specimens. CPPLIP-010 (Metacarpal I) in (A) anterior (external); (B) posterior (internal); (C) lateral; (D) medial; (E) proximal and (F) distal views. Abbreviations: lpc, laterally projected crest; M. ecr, insertion for the *M. extensor* carpi radialis; ppc, posteriorly projected crest.

CPPLIP-010 (right metacarpal I, Fig. 16). Both proximal and distal surfaces of the bone are slightly convex. The first is heavily anteroposteriorly compressed and bears a small posterior projection, whereas the distal surface is subtriangular in distal view. The anterior (external) surface is flat. Distally, the shaft becomes concave laterally and the posterior surface bears a proximodistally oriented crest along the mid-shaft. On the lateral surface, another crest extends longitudinally along the bone. Medially, there is a small concavity where *M. extensor carpi radialis* inserted.

**Figure 17:**
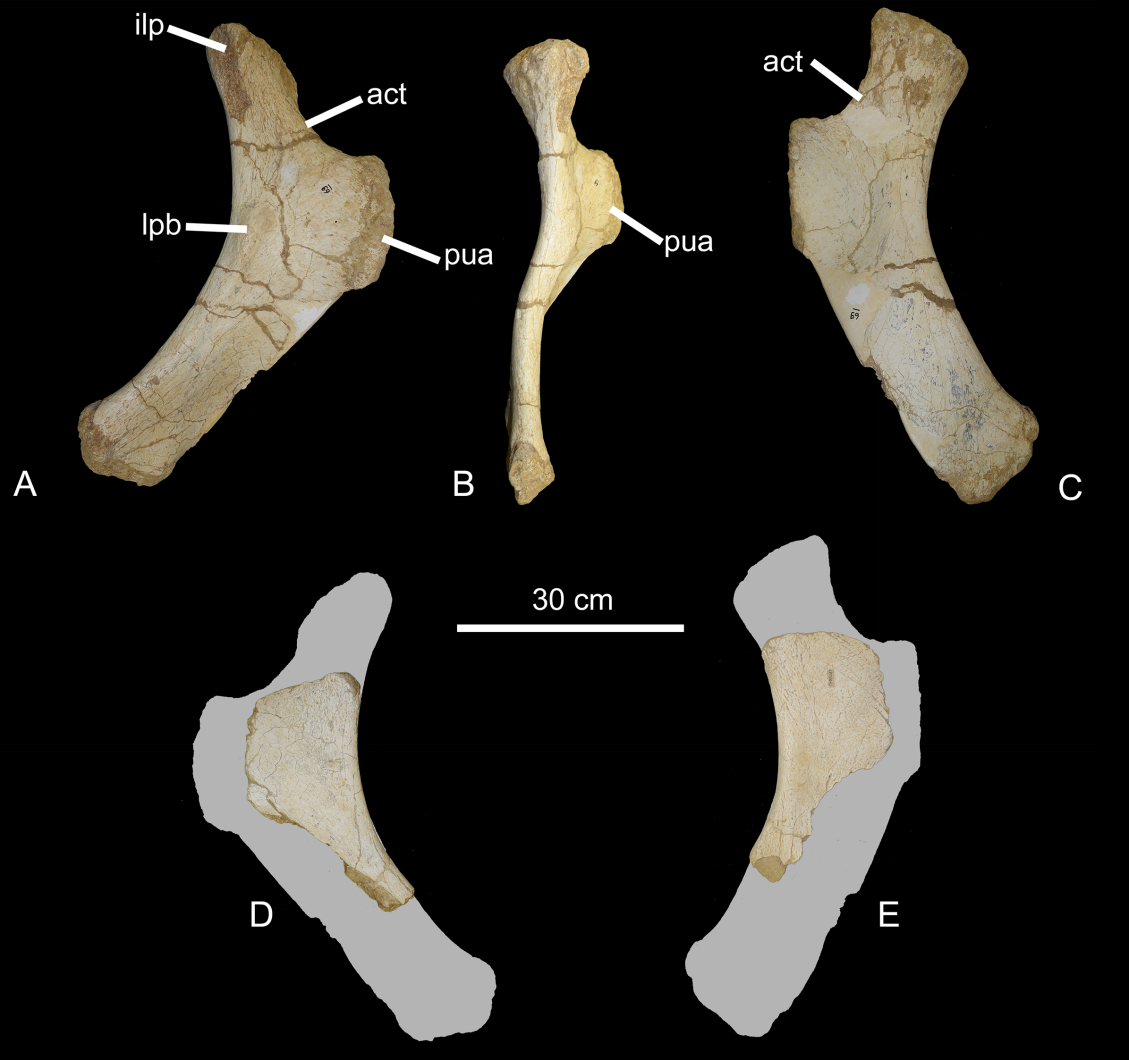
Ischia of the BR-262 specimens. CPPLIP-069 (right ischium) in (A) lateral; (B) dorsal and (C) medial views. CPPLIP-042 (left ischium) in (D) lateral and (E) medial views. Abbreviations: act, acetabulum; lpb, lateral protuberance; ilp, iliac peduncle; pua, pubic articulation.

*Pelvic girdle*. CPPLIP-069 and 042 (right and left ischia, Fig. 17). CPPLIP-069 is complete and well-preserved, whereas CPPLIP-042 has only the proximal portion preserved. The ischium is a gracile element with a strongly concave posterodorsal margin. The contribution to the acetabular margin is *via* a thin, concave lamina. Anterodorsally, the bone expands lateromedially, forming a robust iliac peduncle, that has a rectangular outline in lateral/medial views. The lateral surface bears a lateral protuberance, which represents the attachment of the ischial head of *M. flexor tibialis*. On the anteroventral margin, the bone thickens, forming the pubic articulation. Posterior to that, the ventral margin is formed by a thin lamina. The medial surface of the ischium is mainly flat, with its proximal portion slightly bulged medially, close to the pubic articulation.

**Figure 18:**
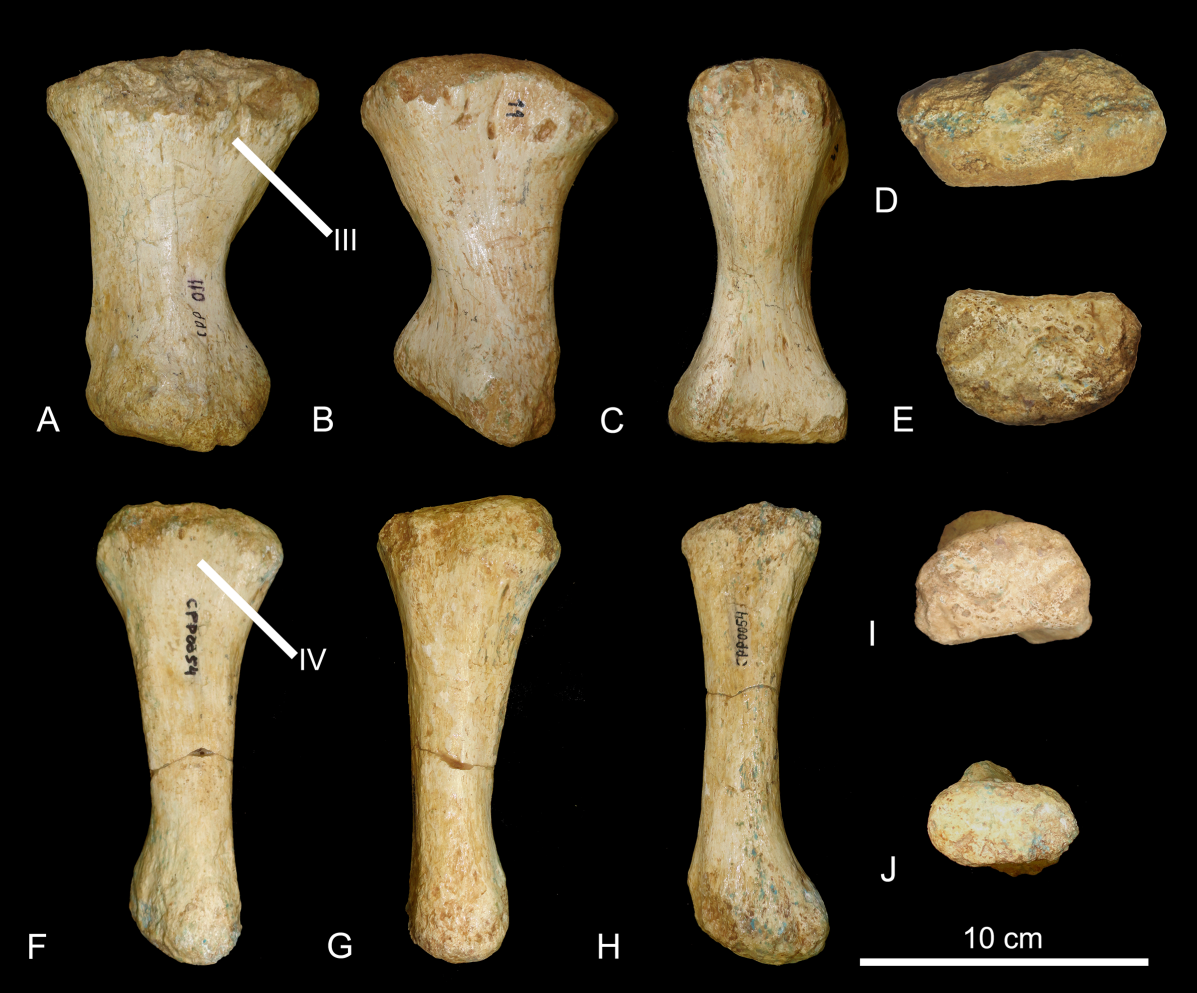
Metatarsals of the BR-262 specimens. CPPLIP-011 (left metatarsal II) in (A) medial; (B) lateral; (C) plantar; (D) proximal and (E) distal views. CPPLIP-054 (left metatarsal III) in (F) medial; (G) lateral and (H) plantar; (I) proximal and (J) distal views. Abbreviations: III, articulation with metatarsal III; IV, articulation with metatarsal IV.

*Hindlimb*. CPPLIP-011 and 054 (left metatarsals II and III, Fig. 18). The position of the metatarsals can be inferred based on the shape of the proximal and distal articular surfaces, compared to those of complete pedes, such as those of the “La Invernada” titanosaur (MUCPv-1533) and *Rapetosaurus krausei* (Riga, Calvo & Porfiri, 2008; Curry Rogers, 2009). The proximal ends are lateromedially expanded. CPPLIP-011 has a robust, lateromedially expanded shaft, whereas CPPLIP-054 is a slender element, both having slightly concave ventral margins. Proximally, small concave surfaces indicate where the metatarsals would articulate with the lateral ones. The distal surfaces are dorsoventrally expanded and have rounded distal outlines.

## Discussion

### Comparisons with the Serra da Galga Formation titanosaurs

In an attempt to identify the BR-262 (“Rodovia” site) specimens, we compared them with the three titanosaur nominal species known for the Serra da Galga Formation, based on the holotypes of *T. pricei* and *Ba. britoi*, and the holotype and referred specimens of *U. ribeiroi* (Salgado & De Souza Carvalho, 2008; Silva Junior et al., 2019).

*Uberabatitan ribeiroi—*The BR-262 cervical vertebrae share a number of anatomical features with those of *U. ribeiroi*, such as a ventrolateral crest on the ventral surface of the centra and a neural spine with a bulbous apex (Silva Junior et al., 2019, fig. 4A), but lack the low (dorsoventrally compressed) neural spine apex of *U. ribeiroi*. The BR-262 cervical vertebrae also lack the unique laminar pattern of *U. ribeiroi*, in which the epipophyseal-prezygapophyseal lamina is composed of a zygapophyseal and a diapophyseal portion (Silva Junior et al., 2019, fig. 4A). Instead, the BR-262 cervical vertebrae (CPPLIP-035, CPPLIP-039; Fig. 3) possess a robust, dorsoventrally expanded postzygodiapophyseal lamina. The anterior BR-262 trunk vertebrae (*i.e*., CPPLIP-110 and CPPLIP-036) show a higher degree of pneumatization compared to those of *U. ribeiroi*. They have pneumatic fossae perforated by several small foramina (CPPLIP-036; Fig. 6) and a deep centroparapophyseal fossa, with accessory laminae (CPPLIP-110, 036; Fig. 6). Instead, *U. ribeiroi* trunk vertebrae have deep pneumatic fossae and centroparapophyseal fossae, but no foramina or accessory laminae (Silva Junior et al., 2019, fig. 7A).

The BR-262 caudal vertebrae (*e.g*., CPPLIP-102; Fig. 10) also differ from those of *U. ribeiroi* (Silva Junior et al., 2019, fig. 9) by lacking strongly excavated lateral surfaces of the centrum and the tubercle on the proximal portion of the transverse processes. The preserved neural spines of the BR-262 tail vertebrae are strongly inclined posteriorly, also differing from those of *U. ribeiroi*, the neural spines of which vary from vertically oriented to only slightly inclined anteriorly (Silva Junior et al., 2019, figs. 9–12).

Both anterior and posterior chevrons of the BR-262 specimens differ from those of *U. ribeiroi*. Its anterior chevrons possess more robust proximal rami (Fig. 13), whereas those of *U. ribeiroi* are mediolaterally flattened (Silva Junior et al., 2019, fig. 14A–14D). The distal rami of *U. ribeiroi* chevrons are also strongly mediolaterally flattened, forming a robust anteriorly projected crest. Only the proximal rami of the posterior chevrons are preserved in the BR-262 specimens (Fig. 13). Those share with *U. ribeiroi* the presence of a laterally projected crest, but this crest is more robust in the latter taxon (Silva Junior et al., 2019, fig. 14E). In addition, *U. ribeiroi* possesses haemal canals with a wider dorsal opening than those of the BR-262 specimens.

*Trigonosaurus pricei* (MCT 1488-R)—The BR-262 cervical vertebrae share similarities with those from the middle-posterior part of the *T. pricei* neck, including a ventrolateral crest and a low neural spine with a bulbous apex, although this apex is located more posteriorly in relation to the centrum than in *T. pricei*. In addition, the mid-posterior cervical vertebrae of *T. pricei* have dorsoventrally expanded postzygodiapophyseal laminae.

**Figure 19:**
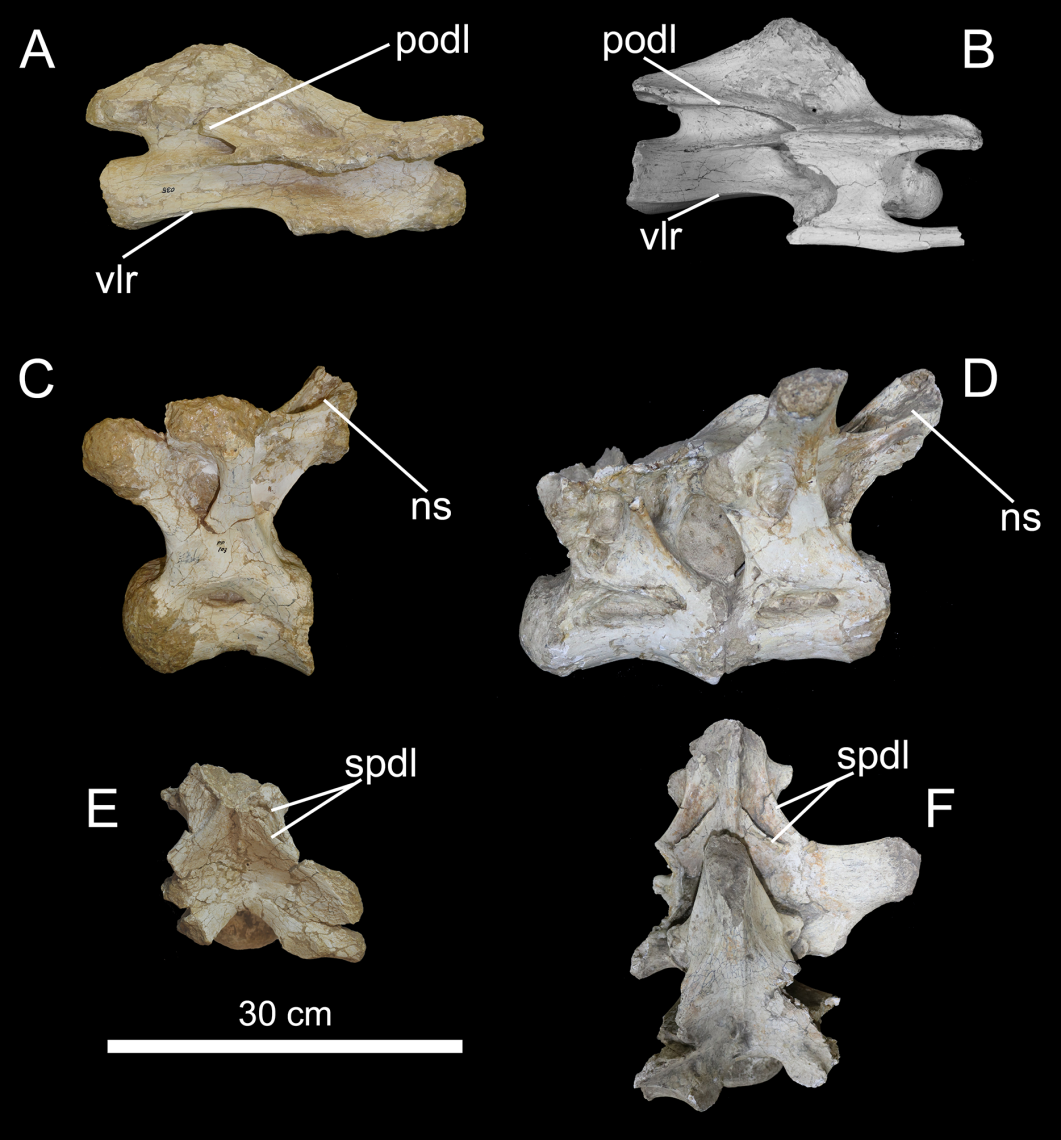
Axial elements of MCT 1488-R and BR-262. (A) Right lateral view of CPPLIP-035. (B) Possible 9^th^ cervical vertebrae of *T. pricei*, left (reversed) lateral view. CPPLIP-103 in (C) left lateral, and (E) dorsal, views. 4^th^ and 5^th^ trunk vertebrae of *T. pricei* in (D) left lateral, and (F) dorsal, views. Abbreviations: ns, neural spine; podl, postzygodiapophyseal lamina; vlr, ventrolateral ridge; spdl, anterior and posterior spinodiapophyseal laminae.

The trunk vertebrae from BR-262 are quite similar to those of *T. pricei*, so that they can be directly compared to the different trunk regions of the latter. CPPLIP-036 and 110 are compatible with the most anterior trunk vertebrae of *T. pricei*. They share large pneumatic fossae—with almost half of the centrum height—and deep postzygapophyseal spinodiapophyseal fossae that extend anteroventrally and are delimited by robust spinodiapophyseal laminae (Fig. 6). CPPLIP-103 and 111 are similar to the middle trunk vertebrae of *T. pricei*. They share neural spines with a strong posterior inclination, so they surpass the margin of the cotyle (CPPLIP-103; Fig. 19), a condition that was tentatively proposed as autapomorphic for *T. pricei* (Campos et al., 2005, fig. 15). Further, their spinodiapophyseal laminae are divided into anterior and posterior portions (CPPLIP-103, Fig. 19; Campos et al., 2005, fig. 18). As for CPPLIP-037 and 458, they are comparable to the most posterior trunk vertebrae of *T. pricei*, sharing pneumatic fossae restricted to the dorsal portion of the centra, ventrally delimiting large centrodiapophyseal fossae (Figs. 8 and 9; Campos et al., 2005, fig. 19). On the other hand, the BR-262 specimens lack the postzygodiapophyseal lamina that laterally connects the postzygapophyses with the diapophyses, which was tentatively proposed as an autapomorphy for *T. pricei* (Campos et al., 2005).

**Figure 20:**
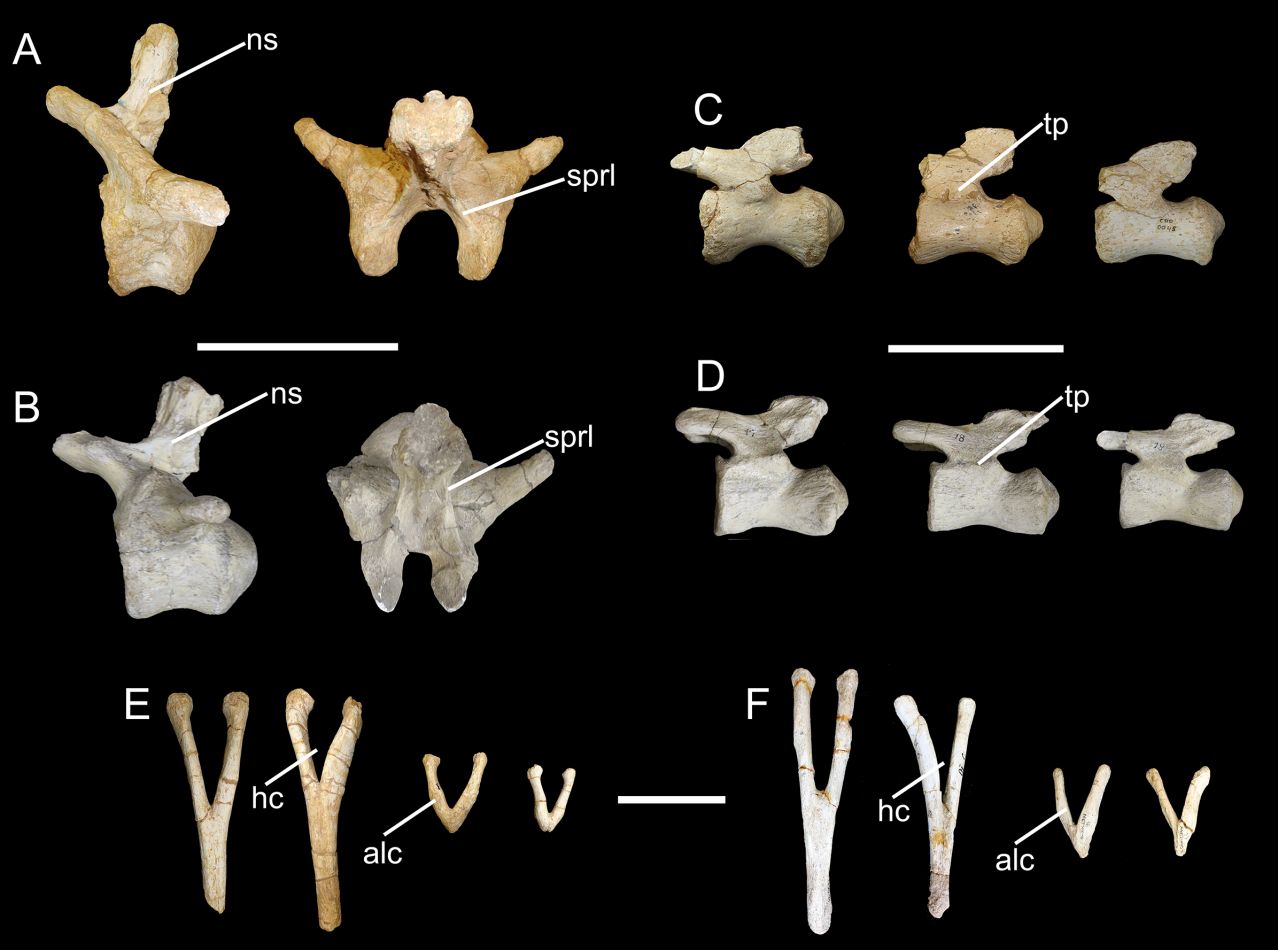
Caudal elements of *Baurutitan britoi* and BR-262 specimens. (A) Anterior caudal vertebra of BR-262 specimens in left lateral and dorsal views. (B) Anterior caudal vertebra of *B. britoi* in left lateral and dorsal views. (C) Mid-posterior caudal vertebrae of BR-262 specimens in left lateral views. (D) Mid-posterior caudal vertebrae of *B. britoi* in left lateral views. (E) Chevrons of BR-262 specimens in anterior view. (F) Chevrons of *B. britoi* in anterior view. Abbreviations: alc, anterolateral projecting crest; hc, haemal canal; ns, neural spines; sprl, spinoprezygapophyseal lamina; tp, transverse process. Scale for anterior vertebrae: 20 cm; scale for mid-posterior vertebrae: 15 cm; scale for chevrons: 10 cm.

*Baurutitan britoi* (MCT 1490-R)—The BR-262 caudal series is quite similar to that of MCT 1490-R. Although the exact position of CPPLIP-102 cannot be defined, it is similar to the most anterior elements of *Ba. britoi*. The 2^nd^ and 3^rd^ caudal vertebrae of *Ba. britoi* possess aEIs of 0.6 and 0.7, respectively, similar to the 0.7 value of CPPLIP-102. They also share neural spines that are posteriorly inclined and slightly curved forwards (Fig. 20; Kellner, Campos & Trotta, 2005, fig. 16), though the neural spines of *Ba. britoi* are displaced more posteriorly in the centra. The prezygapophyses of CPPLIP-102 are also located more laterally than those of *Ba. britoi*.

CPPLIP-046, 047, and 061 are similar to the middle caudal vertebrae of *Ba. britoi*, though their positions cannot be precisely defined. They share non deeply excavated centra and posteriorly inclined neural spines, characters also present in more posterior caudal vertebrae. CPPLIP-093 and 045 seem to be from a more posterior portion of the tail than that preserved in *Ba. britoi* (Fig. 20; Kellner, Campos & Trotta, 2005, fig. 22), so that they are not directly comparable.

The chevrons of the BR-262 specimens (Fig. 13) are also similar to those of *Ba. britoi*. They share dorsally open haemal canals in both anterior and posterior elements. *Baurutitan britoi* also shows proximal rami with laterally projected crests, although this feature is asymmetrically distributed, present in just one of the sides of one of the most posterior chevrons (Kellner, Campos & Trotta, 2005, figs. 26 and 27). A similar laterally projected crest is visible on both right sides of CPPLIP-100 and 055 (Fig. 20), although less prominent on the latter specimens.

In sum, although the BR-262 titanosaur specimens can be differentiated from those referred to *U. ribeiroi*, only very minor differences exist compared to the holotypes of *T. pricei* and *Ba. britoi*. This is further evidenced by the presence of either autapomorphies or unique sets of features of both *Ba. britoi* and *T. pricei*, which are also present in the BR-262 material, as highlighted below.

Campos et al. (2005) identified a set of traits in the cervical vertebrae of MCT 1488-R as autapomorphies of *T. pricei*, including elongated mid-cervical vertebrae, with low neural spines and concave ventral margins. The latter two traits are also seen in the preserved BR-262 cervical elements (Fig. 3). The 9^th^ cervical vertebra of *T. pricei* (Campos et al., 2005, figs. 8–10) and a slightly more anterior cervical vertebra from BR-262 (CPPLIP-035) have both aEIs of 3.4. Here, we also identified a new feature shared uniquely by MCT 1488-R and the BR-262 cervical vertebrae (Fig. 19), namely a robust (*i.e*., dorsoventrally expanded) postzygodiapophyseal lamina. This differs from the condition present in other titanosaurs, in which both the posterior centrodiapophyseal and the postzygodiapophyseal laminae have similar proportions, as seen in *Futalognkosaurus dukei* (Calvo, González Riga & Porfiri, 2007; fig. 2), *Rinconsaurus caudamirus* (Calvo & González Riga, 2003; Plate 2), and *Rapetosaurus krausei* (Curry Rogers, 2009; fig. 9).

Campos et al. (2005) also proposed autapomorphic features for the trunk vertebrae of *T. pricei* (MCT 1488-R): *i.e*., elongated mid-trunk vertebrae, with strongly posteriorly inclined neural spines, and trunk vertebrae 9–10 with incipient postzygodiapophyseal laminae. The anteroposterior length (excluding the condylar ball) to cotyle height ratio in the mid-trunk vertebrae of MCT 1488-R is ~1.3, whereas a lower value (~1.0) is seen in BR-262 specimens. Regarding the neural spines, those of MCT 1488-R form an angle of ~55° to the centrum. Strongly posteriorly inclined neural spines are also seen in BR-262 trunk vertebrae (CPPLIP-103; Fig. 19), but poor preservation precludes a precise measurement of the angle. The vertebrae identified as most posterior of the BR-262 specimens lack such incipient postzygodiapophyseal laminae.

Kellner, Campos & Trotta (2005) identified a couple of features in the holotype of *Ba. britoi* (MCT 1490-R) as potential autapomorphies of that species: *i.e*., strongly pointed and laterally directed process intercepting the spinoprezygapophyseal lamina on the first caudal vertebra and anterolaterally directed spinoprezygapophyseal laminae. A first caudal vertebra cannot be unambiguously identified in the BR-262 specimens, hampering the assessment of the former character, whereas the spinoprezygapophyseal laminae (CPPLIP-102; Fig. 20) are more laterally located in their neural spines than in those of MCT 1490-R.

### Reassessment of *Baurutitan britoi* and *Trigonosaurus pricei*

The taxonomic status of *T. pricei* and *Ba. britoi* has to be analyzed based on some basic premises: 1—Although both species possess one overlapping element, the last sacral vertebra, it disallows any meaningful comparison; 2—The holotypes of both species are anatomically compatible with BR-262 specimens; 3—The BR-262 caudal vertebrae differ from those of MCT 1719-R (paratype of *T. pricei*; see below). Further, there is no *prima facie* evidence that the caudal series MCT 1719-R belongs to *T. pricei*—their association was first rejected by Campos & Kellner (1999) and then accepted based on sparse evidence by Campos et al. (2005)—so two taxonomic scenarios are possible. If the tail MCT 1719-R were assigned to *T. pricei*, then *T. pricei* and *Ba. britoi* could be distinguished based solely on their different caudal vertebrae and the BR-262 material would be assigned to *Ba. britoi* based on the caudal vertebral anatomy, even if its cervical and trunk vertebrae are totally compatible with those of *T. pricei*. On the other hand, if MCT 1719-R is not *a priori* assigned to *T. pricei*, the matching anatomy of the BR-262 specimens to the holotypes of both *T. pricei* (MCT 1488-R) and *Ba. britoi* (MCT 1490-R) indicates that those two taxa are not taxonomically disparate. In this case, the caudal series MCT 1719-R would represent a hitherto undescribed new species, because it is not compatible with either MCT 1490-R or the BR-262 specimens, (see below). We consider the latter arrangement, which results in the synonymization of *T. pricei* and *Ba. britoi* better justified, so that these two species are not differentiated only based on characters found in a specimen ambiguously associated to *T. pricei*.

*Trigonosaurus pricei* and *Ba. britoi* were both first published in the same volume, but nomenclatural priority is given to *Ba. britoi*, because it was proposed some pages ahead (p. 529) of *T. pricei* (p. 565). So, if considered synonyms, as suggested here, *Ba. britoi* is the name to be adopted. Likewise, the set of BR-262 specimens is also referred to *Ba. britoi*, the systematic paleontology of which is given below.

### Systematic paleontology

*Dinosauria* Owen, 1842; Langer et al., 2020

*Sauropodomorpha* Huene, 1932; Fabbri et al., 2020

*Titanosauriformes* Salgado, Coria & Calvo, 1997, Silva Junior et al., 2022

*Titanosauria* Bonaparte & Coria, 1993, Silva Junior et al., 2022

*Baurutitan britoi* Kellner, Campos & Trotta, 2005

*Syn. Trigonosaurus pricei* Campos et al., 2005 (a complete list of synonyms is provided on the supplementary)

***Type-species:*** *Baurutitan britoi* Kellner, Campos & Trotta, 2005

***Holotype:*** MCT 1490-R (Series C): last sacral vertebra articulated with a sequence of eighteen caudal vertebrae.

***Referred specimens:*** MCT 1488-R (Series B; holotype of *T. pricei*): five cervical and 10 trunk vertebrae; sacrum and ilium. Forty-four specimens, possibly constituting a single individual, recovered from BR-262 locality, including: CPPLIP-035 (middle cervical vertebrae), CPPLIP-039 (middle cervical vertebrae), CPPLIP-040 (posterior cervical vertebrae), CPPLIP-049 (posterior cervical vertebrae), CPPLIP-014 (cervical rib), CPPLIP-110 (anterior trunk vertebra), CPPLIP-036 (anterior trunk vertebra), CPPLIP-103 (middle trunk vertebra), CPPLIP-111 (middle trunk vertebra), CPPLIP-037 (middle trunk vertebrae), CPPLIP-458 (middle trunk vertebrae), CPPLIP-43 (posterior trunk neural spine), CPPLIP-044 (trunk rib fragment), CPPLIP-097 (trunk rib fragment), CPPLIP-108 (trunk rib fragment), CPPLIP-109 (trunk rib fragment), CPPLIP-102 (anterior caudal vertebra), CPPLIP-046 (middle caudal vertebra), CPPLIP-047 (middle caudal vertebra), CPPLIP-061 (middle caudal vertebra), CPPLIP-096 (middle caudal vertebra), CPPLIP-091 (posterior caudal vertebra), CPPLIP-093 (middle caudal vertebra), CPPLIP-094 (posterior caudal vertebra), CPPLIP-095 (posterior caudal vertebra), CPPLIP-045 (posterior caudal vertebra), CPPLIP-055 (anterior chevron), CPPLIP-056 (anterior chevron), CPPLIP-098 (anterior chevron), CPPLIP-099 (anterior chevron), CPPLIP-112 (anterior chevron), CPPLIP-188 (anterior chevron), CPPLIP-057 (posterior chevron), CPPLIP-100 (posterior chevron), CPPLIP-038 (right scapula), CPPLIP-140 (right coracoid), CPPLIP-138 (right sternal plate), CPPLIP-007 (fragment of left humerus), CPPLIP-008 (right humerus), CPPLIP-010 (right metacarpal I), CPPLIP-042 (left ischium fragment), CPPLIP-069 (right ischium), CPPLIP-011 (left metatarsal II), CPPLIP-054 (left metatarsal III).

***Type-locality and horizon:*** MCT 1490-R was collected from the Serra da Galga Formation (Soares et al., 2021), in the site known as “Caieira”, “Quarry 1”, or “Ponto 1 do Price”, Serra do Veadinho area, near Peirópolis, Uberaba-MG (Campos & Kellner, 1999; Martinelli & Teixeira, 2015).

***Revised diagnosis:*** titanosaur diagnosed based on a set of autapomorphic features, *i.e*.: expanded postzygodiapophyseal laminae on mid-posterior cervical vertebrae (newly proposed here) and first caudal vertebra with strongly pointed and laterally directed processes intercepting the spinoprezygapophyseal lamina (Kellner, Campos & Trotta, 2005).

**Reassessment of MCT 1719-R**

**Figure 21:**
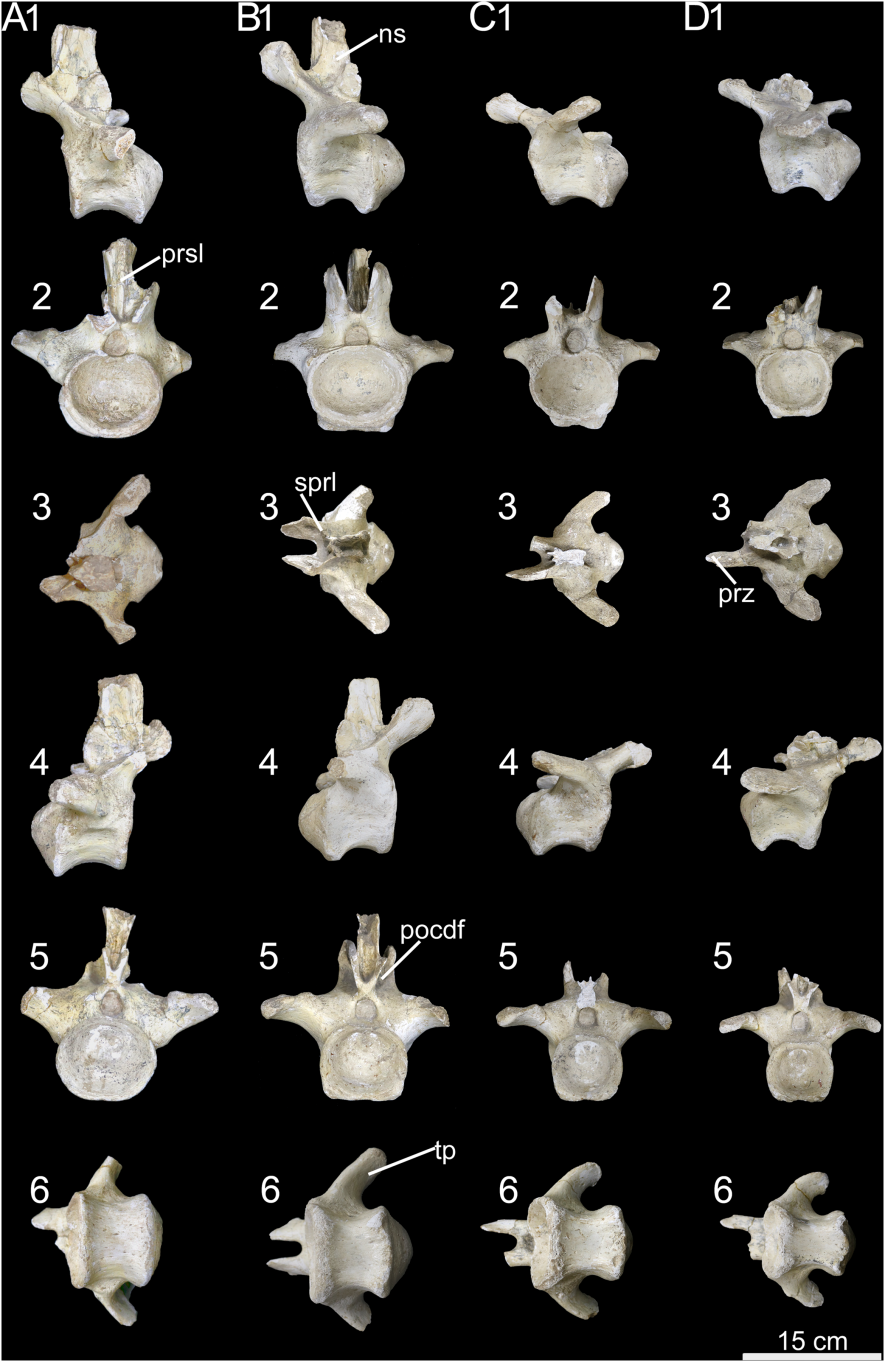
Anterior caudal vertebrae of *Caieiria allocaudata* (MCT 1719-R). In (1) left lateral; (2) anterior; (3) dorsal; (4) right lateral; (5) posterior and (6) ventral views. Abbreviations: ns, neural spine; pocdf, postzygapophyseal centrodiapophyseal fossa prz, prezygapophyses; sprl, spinoprezygapophyseal lamina; tp, transverse process.

**Figure 22:**
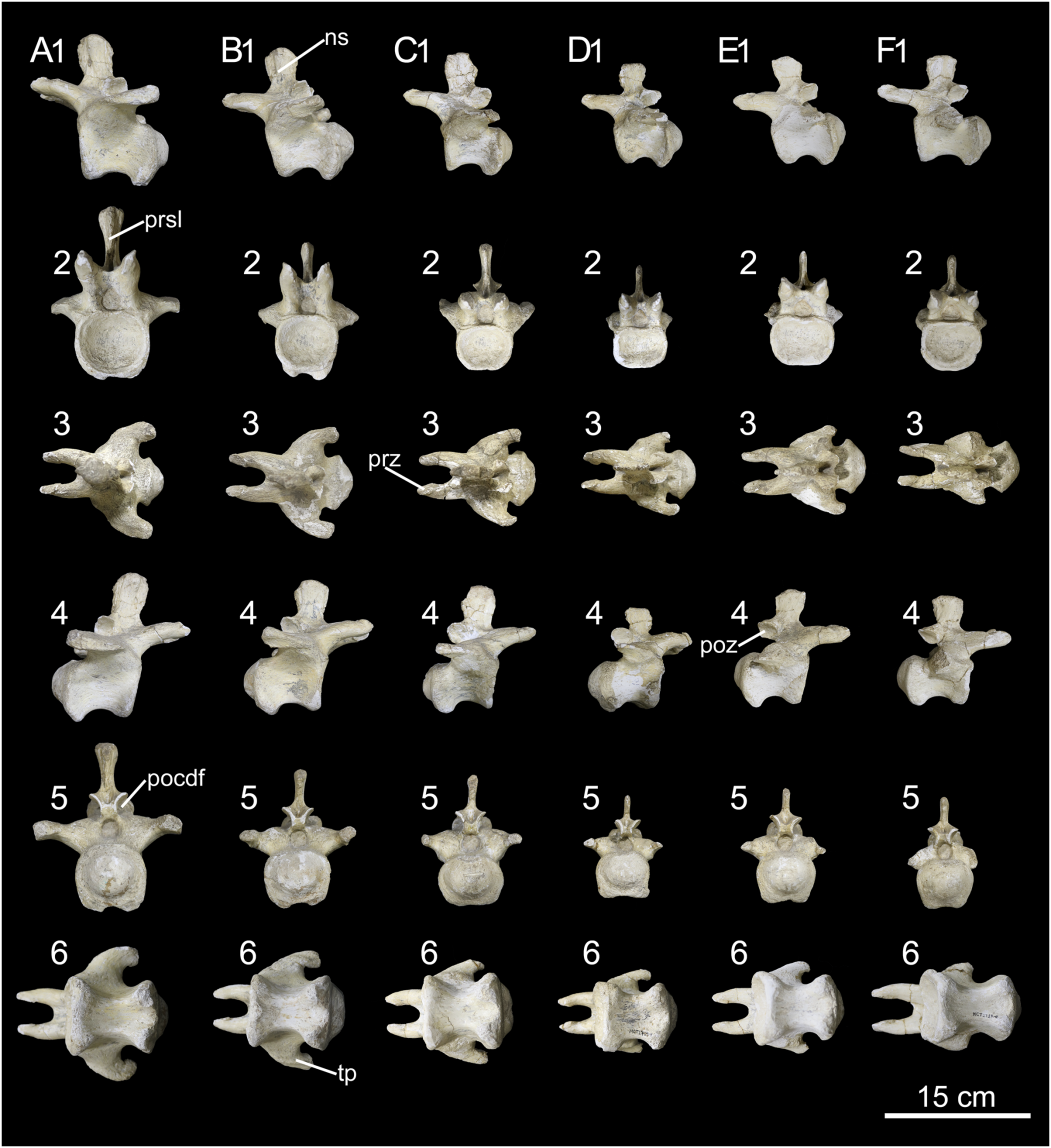
Middle caudal vertebrae of *Caieiria allocaudata*. In (1) left lateral; (2) anterior; (3) dorsal; (4) right lateral; (5) posterior and (6) ventral views. Abbreviations: ns, neural spine; pocdf, postzygapophyseal centrodiapophyseal fossa; poz, postzygapophyses; prz, prezygapophyses; prsl, prespinal lamina; tp, transverse process.

The redefinition of the specimens referred to *Ba. britoi* implies that MCT 1719-R cannot be associated to that taxon, as these caudal vertebrae clearly differ from those of MCT 1490-R and the BR-262 specimens. As discussed above, the BR-262 caudal neural spines lean posteriorly, as also seen in *Ba. britoi* (Kellner, Campos & Trotta, 2005, figs. 8, 12, 16 and 19), but not in MCT 1719-R, the spines of which lean gently anteriorly or stand nearly vertical (Figs. 21, 22). MCT 1719-R also lacks another trait shared between *Ba. britoi* and the BR-262 specimens: transverse processes that turn into a lateral ridge on the middle of the series. Below, we further revise the features of MCT 1719-R that Campos et al. (2005) used to diagnose *T. pricei*.

Campos et al. (2005) proposed that the centra of the anterior tail vertebrae possess thin ventral margins that broaden towards the top and transverse processes with pronounced dorsal depressions, two in the anterior (2–5) and one in the middle caudal vertebrae. The 2^nd^ caudal vertebra possesses a deep muscular scar on its lateral face, followed by centra with lateral faces more deeply excavated than those at a similar serial position in *Gondwanatitan faustoi* (Kellner & Azevedo, 1999; fig. 6), *Panamericansaurus schroederi* (Porfiri & Calvo, 2010; fig. 3), and *U. ribeiroi* (Silva Junior et al., 2022; fig. 10). Also, anteriorly extended caudal prezygapophyses, with wide (dorsoventrally expanded) articular faces, are unique to MCT 1719-R among titanosaurs from the Serra da Galga Formation. These are about 70% the centrum length in middle caudal vertebrae, a proportion similar to that found on some *Aeolosaurini*, such as *Aeolosaurus rionegrinus* (72%; Powell, 1987) and *Arrudatitan maximus* (76%; Santucci & Arruda-Campos, 2011). The latter also shares wide articular facets (Santucci & Arruda-Campos, 2011; fig. 4) with MCT 1719-R, as well as with *Punatitan coughlini* (Hechenleitner et al., 2020).

As mentioned by Campos et al. (2005), MCT 1719-R has articular surfaces for the haemal arches that are strongly developed from the third caudal vertebra until the last preserved element (20^th^ caudal vertebra). Although suggested as a unique feature of MCT 1719-R, a similar condition is present in *Rocasaurus muniozi* (Salgado & Azpilicueta, 2000; figs. 6 and 8) and *U. ribeiroi* (Silva Junior et al., 2022; fig. 10). Finally, the presence of well-developed transverse processes along the anterior and middle (1–20) caudal vertebrae was also proposed as unique to MCT 1719-R (Campos et al., 2005). In fact, some other titanosaurs—*e.g*., *Ar. maximus* (Santucci & Arruda-Campos, 2011; fig. 4) and *U. ribeiroi* (Silva Junior et al., 2022; fig. 9)—possess transverse processes as long as those of MCT 1719-R (Figs. 21 and 22), almost reaching the posterior margin of the condyles, although less developed in more posterior vertebrae. Yet, those of MCT 1719-R are unique because they are strongly expanded dorsoventrally, to almost half the centrum height, including those of middle caudal vertebrae. As for the persistence of the transverse processes minimally until the twentieth caudal vertebra; this feature is also present in *Overosaurus paradosorum* (Coria et al., 2013; fig. 6) and *P. coughlini* (Hechenleitner et al., 2020; fig. 2).

Our comparative review has shown the presence of yet another unique feature of MCT 1719-R: the presence of deep postzygapophyseal-centrodiapophyseal fossae, expanding anteromedially on the dorsal margin of the neural arch (Figs. 21, 22). This condition differs from that of other titanosaurs, in which this fossa is present but does not expand medially, as for instance in *Ba. britoi* (Fig. 20D: Kellner, Campos & Trotta, 2005; fig. 18), *U. ribeiroi* (Silva Junior et al., 2019; fig. 9), and the BR-262 specimens. A well-developed postzygapophyseal-centrodiapophyseal fossa is also present in *Adamantisaurus mezzalirai* (Santucci & Bertini, 2006; plate 1), but restricted to the most anterior vertebrae and not as deep as in MCT 1719-R. Deep postzygapophyseal-centrodiapophyseal fossae are also present in *Narambuenatitan palomoi* (Filippi, García & Garrido, 2011; fig. 8) and *Mendozasaurus neguyelap* (González Riga et al., 2018; fig. 9), although these are dorsoventrally expanded in the former, reaching the neural canal, and limited medially by a centropostzygapophyseal lamina in the latter.

In conclusion, the uniqueness of MCT 1719-R among Bauru Group and other South American titanosaurs, including the presence of autapomorphic features (see below), warrants the proposition of a new taxon to accommodate the specimen.

### Systematic paleontology

*Dinosauria* Owen, 1842; Langer et al., 2020

*Sauropodomorpha* Huene, 1932; Fabbri et al., 2020

*Titanosauriformes* Salgado, Coria & Calvo, 1997, Silva Junior et al., 2022

*Titanosauria* Bonaparte & Coria, 1993, Silva Junior et al., 2022

*Caieiria allocaudata* gen. et sp. nov.

***Etymology:*** The generic name derives from “Caieira”, the site where the type-specimen was unearthed. The specific name employs the word *allos* (Greek for strange) and cauda (Latin for tail), in reference to the unique anatomy of the animal’s tail vertebrae.

***Holotype:*** MCT 1719-R, 10 anterior to middle caudal vertebrae.

***Type-locality and horizon:*** MCT 1719-R was collected in the site known as “Caieira”, or “Quarry 1”, Serra do Veadinho area, near Peirópolis, Minas Gerais, Brazil (Campos & Kellner, 1999). The bearing sandstones belong to the Serra da Galga Formation, Bauru Group (Martinelli et al., 2019; Soares et al., 2020, 2021).

***Diagnosis***: *Caieiria allocaudata* can be distinguished from *Baurutitan britoi, Uberabatitan ribeiroi*, and *Gondwanatitan faustoi* by the presence of caudal vertebrae with robust and dorsoventrally expanded transverse processes, almost half the centrum height (modified from Campos et al., 2005), and anterior caudal vertebrae with a deep postzygapophyseal centrodiapophyseal fossa (newly proposed here).

**Phylogenetic analysis**

**Figure 23:**
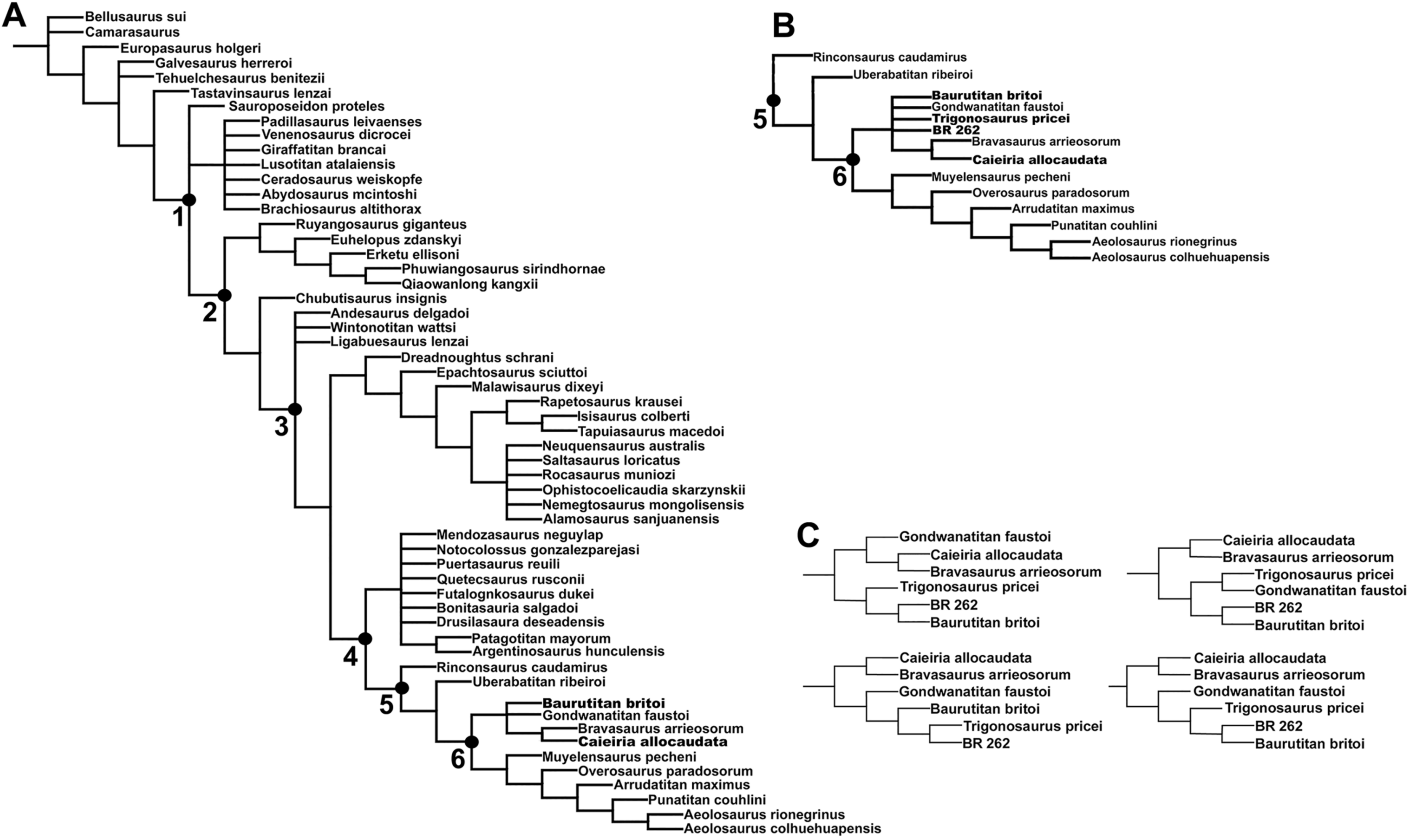
Phylogenetic results. (A) Strict consensus of the 1,500 MPTs found in the second iteration; (B) simplified strict consensus of the 1,620 MPTs found in the first iteration. (C) Alternative arrangements for the Serra da Galga Specimens and *G. faustoi* on Iteration I. Nodes: 1, Titanosauriformes; 2, Somphospondylii; 3, Titanosauria; 4, Colossosauria; 5, Rinconsauria; 6, Aeolosaurini.

For the first iteration we added the BR-262 specimens, plus the holotypes of *Ba. britoi*, *T. pricei*, and *C. allocaudata* to the matrix. This resulted in 1,620 most parsimonious trees (MPTs) of 1,504 steps. The strict consensus tree (Fig. 23B) shows *Gondwanatitan faustoi*, the BR-262 specimens, plus the holotypes of *Ba. britoi* and *T. pricei*, within a polytomy along with a clade including *C. allocaudata* and *Bravasaurus arrierosorum*. In the entire set of MPTs, four possible arrangements for this polytomy were found, as seen in Fig. 23C. *Caieira allocaudata* and *Br. arrierosorum* form a minimal clade in all alternative arrangements, sister to either *G. faustoi* or to a clade congregating the other Serra da Galga Formation titanosaurs. Alternatively, *G. faustoi* was recovered either within or as sister-taxon to the specimens assigned here to *Ba. britoi*.

The second iteration was performed with the coding of the BR-262 specimens and the holotypes of *Ba. britoi* and *T. pricei* combined. This resulted in 1,500 MPTs of 1,502 steps. The strict consensus tree (Fig. 23A) shows *Ba. britoi* in a polytomy with *G. faustoi* and a clade including *C. allocaudata* and *Br. arrierosorum*. The clade congregating these four taxa is supported by a single synapomorphy: middle to posterior trunk vertebrae with pneumatic fossae located on the dorsal margin of the centra (Ch. 189), as seen in *Ba. britoi* and *Br. arrierosorum*. The clade composed of *C. allocaudata* and *Br. arrierosorum* is also united by a single synapomorphy: posteriormost anterior and middle caudal vertebrae with vertical neural spines (Ch. 257).

With additional specimens (MCT 1488-R and BR-262), the phylogenetic results confirm the position of *Ba. britoi* as an *Aeolosaurini*, as proposed by Hechenleitner et al. (2020) and Silva Junior et al. (2022). Previously, *Ba. britoi* was recovered either as a *Lithostrostia* indet. (Carballido et al., 2017; Filippi, Salgado & Garrido, 2019) or as a Saltasaurinae-like taxon (*e.g*., Santucci & Arruda-Campos, 2011; França et al., 2016; Gorscak et al., 2017; Carballido et al., 2020). As for the now defunct *T. pricei*, besides its recent association to *Aeolosaurini* (Hechenleitner et al., 2020; Silva Junior et al., 2022), it has been previously recovered in disparate positions within *Lithostrotia* (*e.g*., Bandeira et al., 2016; Martínez et al., 2016; Gorscak & O’Connor, 2019).

The affinity of *C. allocaudata* also to *Aeolosaurini* reinforces that this clade dominated the Late Cretaceous sauropod fauna of the Bauru Basin. This is the case not only of the Serra da Galga Formation, with *Ba. britoi*, *U. ribeiroi*, and *C. allocaudata*, but also of the Adamantina Formation, with *Ar. maximus* and *G. faustoi* (Santucci & Arruda-Campos, 2011; Silva Junior et al., 2022).

### Comparisons to closely related taxa

Apart from the uniqueness of *Ba. britoi* and *C. allocaudata* established here on anatomical/phylogenetic grounds, both taxa also differ from the closely related *G. faustoi* and *Br. arrierosorum*. *Baurutitan britoi* and *G. faustoi* differ because the latter possesses trunk vertebrae with short condyles that are more ventrally displaced, surpassing the ventral margin of the centra (Kellner & Azevedo, 1999; fig. 7), and a humerus that is less mediolaterally expanded and slightly more medially curved (Kellner & Azevedo, 1999; fig. 20) than that of *Ba*. *britoi*. *Baurutitan britoi* and *C. allocaudata* caudal vertebrae differ from those of *G. faustoi* because the latter have neural arches located on the anterior margin of the centra, with long prezygapophyses that exceed the centrum length (Kellner & Azevedo, 1999; Figs. 11 and 12).

*Baurutitan britoi* differs from *Br. arrierosorum* because the middle posterior cervical vertebrae of the latter lack ventrolateral crests projecting from the centra. Middle caudal vertebrae of *Ba. britoi* differ from those of *Br. arrierosorum*, because the latter lacks posteriorly inclined neural spines. Also, those of *Br. arrierosorum* differ from the condition in *C. allocaudata* in the absence of laterally excavated centrum surfaces and in having condyles with posteriorly projected articular surfaces (Hechenleitner et al., 2020; figs. 3h, 3i).

*Baurutitan britoi* has middle cervical vertebrae with neural spines that are lower than those of *Muyelensaurus pecheni* (Calvo et al., 2007; fig. 5) and *Overosaurus paradosorum* (Coria et al., 2013; fig. 2). Also, its trunk vertebrae lack both the ventral crest present in the latter taxon (Coria et al., 2013; fig. 3) and the anteroposteriorly compressed neural spine present in *Punatitan coughlini* (Hechenleitner et al., 2020; fig. 2). The caudal vertebrae of *Ba. Britoi* can be differentiated from those of *Aeolosaurus* spp. And *Arrudatitan maximus*, because they lack the anteriorly located neural arch present in the former (Powell, 1987; fig. 1. and Casal et al., 2007; fig. 2) and the elongated prezygapophyses with expanded facets of the latter taxon (Santucci & Arruda-Campos, 2011; fig. 4). Also, *Ba. Britoi* lacks the strongly posteriorly inclined caudal neural spines present in *M. pecheni* (Calvo et al., 2007; figs. 9, 10) and the crest on the ventral surface of the caudal vertebrae of *O. paradasorum* (Coria et al., 2013; fig. 6).

The caudal vertebrae of *C. allocaudata* lack the anteriorly located neural arch present in *Aeolosaurus* spp. (Powell, 1987; figs. 1. And Casal et al., 2007; fig. 2), and the anteriorly inclined neural spines present in both *Ar. maximus* (Santucci & Arruda-Campos, 2011; fig. 4) and *P. coughlini* (Hechenleitner et al., 2020; fig. 2). *Caieiria allocaudata* also lacks the dorsoventrally expanded neural spines of *M. pecheni* (Calvo, González Riga & Porfiri, 2007; figs. 9, 10) and the ventral crest on the caudal vertebrae of *O. paradasorum* (Coria et al., 2013; fig. 6).

## Conclusions

The description of the titanosaur material unearthed at BR-262 site (Serra da Galga Formation, Bauru Group) shows that it shares several traits with two species previously known from this area and geological unit: *Ba. britoi* and *T. pricei*. A taxonomic revision indicates that *T. pricei* is a junior synonym of *Ba. britoi*, and that the BR-262 specimens belong to that latter species. Our taxonomic revision also revealed that the paratype of *T. pricei* (MCT 1719-R), a caudal vertebral series, actually represents a different species, named here as *Caieiria allocaudata*.

## Supplemental Information

10.7717/peerj.14333/supp-1 Supplemental Information 1 Supplementary material for NEW SPECIMENS OF *BAURUTITAN BRITOI* AND REASSEMENT OF *TRIGONOSAURUS PRICEI*, TWO TITANOSAUR DINOSAURS (SAUROPODA) FROM THE LATE CRETACEOUS OF BRAZIL.

10.7717/peerj.14333/supp-2 Supplemental Information 2 Matrix for Iteration I.

10.7717/peerj.14333/supp-3 Supplemental Information 3 Permission for image use. Signed permission to use a third party image.

10.7717/peerj.14333/supp-4 Supplemental Information 4 Matrix for Iteration 2.

## Institutional abbreviations

CPPLIP: Centro de Pesquisas Paleontológicas Llewellyn Ivor Price, Universidade Federal do Triângulo Mineiro, Uberaba, Brazil
MCT: Museu de Ciências da Terra, Serviço Geológico do Brasil, Rio de Janeiro, Brazil
MUCPv: Museo de Geología y Paleontología Universidad, Nacional del Comahue, Argentina

## Additional Information and Declarations

### Data Availability

The following information was supplied regarding data availability: The table of measurements, referred specimens, coding and the data matrix are available in the Supplemental Files.

### New Species Registration

The following information was supplied regarding the registration of a newly described species: Publication LSID: urn:lsid:zoobank.org:pub:28423C0B-A3E2-4ABF-8751-2E3A8FA98D4A *Caieiria allocaudata* LSID: urn:lsid:zoobank.org:act:7E981FB6-6165-4851-B65A-C3C588FD9021
