# Supplementary material for: New specimens of Baurutitan britoi and a taxonomic reassessment of the titanosaur dinosaur fauna (Sauropoda) from the Serra da Galga Formation (Late Cretaceous) of Brazil
Source: PeerJ. 2022 Nov 15;10:e14333. doi: 10.7717/peerj.14333 (PMC9673870; doi:10.7717/peerj.14333)
Supplement: Supplemental Information 1 [file peerj-10-14333-s001.docx]

**Supplementary material for**

**NEW SPECIMENS OF BAURUTITAN BRITOI AND A TAXONOMIC REASSESSMENT OF THE TITANOSAUR DINOSAUR FAUNA (SAUROPODA) FROM THE SERRA DA GALGA FORMATION (LATE CRETACEOUS) OF BRAZIL**

Julian C. G. Silva Junior^1^, Agustín G. Martinelli^2^, Thiago S. Marinho^3,4^, João I. da Silva^3,5^ and Max C. Langer^1^

^1^ Laboratório de Paleontologia de Ribeirão Preto, Faculdade de Filosofia, Ciências e Letras de Ribeirão Preto, Universidade de São Paulo, Ribeirão Preto, São Paulo, Brazil.

^2^ Sección Paleontología de Vertebrados, Museo Argentino de Ciencias Naturales “Bernardino Rivadavia” – CONICET, Buenos Aires, Argentina.

^3^ Centro de Pesquisas Paleontológicas L. I. Price, Complexo Cultural e Científico Peirópolis, Pró-Reitoria de Extensão Universitária, Universidade Federal do Triangulo Mineiro, Uberaba, Minas Gerais, Brazil.

^4^ Departamento de Ciências Biológicas, Instituto de Ciências Exatas, Naturais e Educação, Universidade Federal do Triângulo Mineiro, Uberaba, Minas Gerais, Brazil.

^5^ Fundação Cultural de Uberaba, Prefeitura municipal de Uberaba, Uberaba, Minas Gerais, Brazil.

**This supplementary file contains:**

| APPENDIX I. Measurements of BR-262 specimens | 02 |
| --- | --- |
| APPENDIX II. List of synonyms of *Baurutitan britoi* | 05 |
| APPENDIX III. References cited in the Supplementary | 07 |
| APPENDIX IV. Scorings for *Baurutitan britoi* and *Caieiria allocaudata* | 16 |

**APPENDIX I. Measurements of BR-262 specimens.**

**Table 1.** Measurements (cm) of cervical vertebrae. ***** = incomplete values; **----** = structure not preserved. **aEI**: average elongation index; **AMCH**: anterior maximum centrum height; **AMCW**: anterior maximum centrum width; **ML1**: maximum length (with condyle); **ML2**: maximum length (without condyle); **NSH**: neural spine height; **PMCH**: posterior maximum centrum height; **PMCW**: posterior maximum centrum width.

| Specimen | aEI | ML1 | ML2 | AMCH | AMCW | PMCH | PMCW | NSH |
| --- | --- | --- | --- | --- | --- | --- | --- | --- |
| CPPLIP-035 | 3,8 | 34,95 | 31,78 | 6,75 | 6,57 | 7,9 | 10,79 | 10,94 |
| CPPLIP-039 | ---- | 22,65* | ---- | ---- | ---- | 5,92 | 8,83 | 8,56* |
| CPPLIP-040 | ---- | 15,42* | ---- | 5,61 | 6,66 | ---- | ---- | ---- |
| CPPLIP-049 | ---- | 18,73* | ---- | 5,55 | 4,57 | ---- | ---- | ---- |

**Table 2.** Measurements (cm) of trunk vertebrae. ***** = incomplete values; **----** = structure not preserved. **AMCH**: anterior maximum centrum height; **AMCW**: anterior maximum centrum width; **ML1**: maximum length (with condyle); **ML2**: maximum length (without condyle); **NSH**: neural spine height; **PMCH**: posterior maximum centrum height; **PMCW**: posterior maximum centrum width.

| Specimen | ML1 | ML2 | AMCH | AMCW | PMCH | PMCW | NSH |
| --- | --- | --- | --- | --- | --- | --- | --- |
| CPPLIP-036 | 12,96 | 9,37 | 6,13 | 11,42 | 7,85 | 10,91 | ---- |
| CPPLIP-037 | 15,98 | 12,82 | 7,82 | 9,99 | 9,32 | 10,65 | 13,68* |
| CPPLIP-043 | ---- | ---- | ---- | ---- | ---- | ---- | 12,79* |
| CPPLIP-103 | 15,85 | 12,52 | 7,73 | 11,23 | 12,83 | 13,41 | ---- |
| CPPLIP-110 | 12,69 | 11,77 | 7,50* | 12,27 | 8,38 | 9,77 | ---- |
| CPPLIP-111 | 16,82 | 11,50 | 9,15* | 10,49* | 9,82* | 11,83 | ---- |
| CPPLIP-458 | 10,49 | 8,61 | 6,22 | 7,81 | 6,3 | 9,01 | 18,68 |

**Table 3.** Measurements (cm) of caudal vertebrae. ***** = incomplete values; **----** = structure not preserved. **aEI:** average elongation index; **AMCH**: anterior maximum centrum height; **AMCW**: anterior maximum centrum width; **ML1**: maximum length (with condyle); **ML2**: maximum length (without condyle); **NSH**: neural spine height; **PMCH**: posterior maximum centrum height; **PMCW**: posterior maximum centrum width.

| Specimen | aEI | ML1 | ML2 | AMCH | AMCW | PMCH | PMCW | NSH |
| --- | --- | --- | --- | --- | --- | --- | --- | --- |
| CPPLIP-045 | 1,6 | 10,81 | 9,19 | 5,06 | 6,28 | 4,7 | 6,63 | 2,09* |
| CPPLIP-046 | 1,2 | 12,31 | 9,4 | 6,34 | 7,43 | 6,71 | 9,14 | ---- |
| CPPLIP-047 | 1,1 | 11,34 | 7,74 | 4,14 | 7,13 | 5,12 | 7,56 | ---- |
| CPPLIP-061 | 0,9 | 10,12 | 7,56 | 5,97 | 6,95 | 7,08 | 8,9 | ---- |
| CPPLIP-091 | 1,8 | 13,44 | 11,37 | 5,55 | 7,68 | 5,41 | 7,32 | ---- |
| CPPLIP-093 | 1,5 | 10,2 | 9,37 | 5,07 | 6,28 | 5,06 | 6,97 | 2,61* |
| CPPLIP-094 | 1,9 | 13,43 | 11,96 | 5,4* | 6,27 | 5,59 | 6,8 | ---- |
| CPPLIP-095 | 1,6 | 12,55 | 9,77 | 5,58* | 7,32 | 4,71 | 7,15 | ---- |
| CPPLIP-096 | 1,7 | 11,51 | 10,06 | 5,93 | 5,75 | 5,4 | 6,45 | 2,44* |
| CPPLIP-102 | 0,7 | 12,72 | 8,32 | 9,45 | 12 | 11,45 | 13,27 | 13,84 |

**Table 4.** Measurements (cm) of chevrons. ***** = incomplete values; **----** = structure not preserved.

| Specimen | Total height | Proximal rami height | Distal rami height |
| --- | --- | --- | --- |
| CPPLIP-055 | 21,95* | 8,86 | 13,09* |
| CPPLIP-056 | 14,53* | 4,12* | 10,41* |
| CPPLIP-057 | ---- | 6,30* | ---- |
| CPPLIP-098 | 22,66 | 9,03 | 13,66 |
| CPPLIP-099 | 20,33* | 11,29 | 9,04* |
| CPPLIP-100 | ---- | 7,31 | ---- |
| CPPLIP-109 | 21,35* | 6,3 | 15,05* |
| CPPLIP-112 | 22,89* | 10,75 | 12,14* |

**Table 5.** Measurements (cm) of pectoral girdle elements. ***** = incomplete values.

| Specimen | Maximum proximodistal length | Maximum mediolateral breadth |
| --- | --- | --- |
| CPPLIP-038 | 64,67 | 44,59 |
| CPPLIP-138 | 46,19 | 23,89 |
| CPPLIP-140 | 26,83* | 24,81* |

**Table 6.** Measurements (cm) of forelimb elements. ---- = structure not preserved; * = incomplete measures. **DAW**: distal anteroposterior width; **DWI**: distal width index; **ECC**: eccentricity index; **ML**: maximum proximodistal Length; **MPTB**: maximum proximal transverse breadth; **MMB**: midshaft mediolateral breadth; **MSC**: midshaft circumference; **MDTB**: maximum distal transverse breadth; **PAW**: proximal anteroposterior width; **PWI**: proximal width index; **RI**: robustness index.

| Specimen | DAW | DWI | ECC | ML | MPTB | MMB | MSC | MDTB | PAW | PWI | RI |
| --- | --- | --- | --- | --- | --- | --- | --- | --- | --- | --- | --- |
| CPPPLIP-007 | ---- | ---- | ---- | 47,86* | 32,5 | ---- | ---- | ---- | 10,83* | ---- | ---- |
| CPPPLIP-008 | 28,62 | 0,28 | 1,2 | 94,63 | 30,28 | 13,18 | 38,6 | 26,83 | 16,57 | 0,31 | 4,03 |
| CPPPLIP-010 | 7,59 | 0,40 | 1,47 | 26,94 | 10,31 | 5,15 | 14,2 | 11,02 | 4,61 | 0,38 | 3,05 |
| CPPPLIP-263 (not described on the manuscript) | ---- | ---- | ---- | 64,09* | 41,44 | ---- | ---- | ---- | 23,62 | ---- | ---- |

**Table 7.** Measurements (cm) of pelvic girdle elements. * = incomplete measures.

| Specimen | Maximum proximodistal length | Maximum mediolateral breadth |
| --- | --- | --- |
| CPPLIP-042 | 33,98* | 14,50* |
| CPPLIP-069 | 64,57 | 16,83 |

**Table 8.** Measurements (cm) of pedal elements. **ML**: maximum proximodistal length, **MPTB**: maximum proximal transverse breadth, **MSB**: maximum shaft breadth, **MDTB**: maximum distal transverse breadth.

| Specimen | ML | MPTB | MSB | MDTB |
| --- | --- | --- | --- | --- |
| CPPLIP-011 | 13,11 | 8,9 | 4,48 | 6,88 |
| CPPLIP-054 | 15,78 | 6,29 | 3,12 | 5 |

**APPENDIX II. List of synonyms of *Baurutitan britoi*.**

A complete list of synonyms is provided here. Following the Open Nomenclature proposed by Matthews, 1973.

.2006 *Baurutitan britoi* Kellner et al.

p.2006 *Baurutitan britoi* Santucci and Bertini

p.2006 *Trigonosaurus pricei* Salgado et al.

.2007 *Baurutitan britoi* Casal et al.

p.2008 *Baurutitan britoi* Salgado and Carvalho

.2009 *Baurutitan britoi* Barco

.2010 *Baurutitan britoi* Calvo and Porfiri

.2010 *Baurutitan britoi* Csiki et al.

p. 2010 *Trigonosaurus pricei* Csiki et al.

.2010 *Baurutitan britoi* García et al.

.2010 *Trigonosaurus pricei* Gallina

.2011 *Baurutitan britoi* D’emic and Wilson

.2011 *Trigonosaurus pricei* D’emic and Wilson

.2011 *Baurutitan britoi* Filippi et al.

.2011 *Trigonosaurus pricei* Filippi et al.

.2011 *Baurutitan britoi* Gallina

.2011 *Trigonosaurus pricei* Gallina

p.2011 *Baurutitan britoi* Kellner et al.

p.2011 *Trigonosaurus pricei* Martinelli et al.

p.2011 *Baurutitan britoi* Santucci and Arruda Campos

p.2012 *Trigonosaurus pricei* Mannion et al.

p.2013 *Trigonosaurus pricei* Carballido and Sander

p.2013 *Trigonosaurus pricei* Coria et al.

.2013 *Baurutitan britoi* D’emic et al.

.2013 *Baurutitan britoi* Filippi et al.

.2013 *Baurutitan britoi* Machado et al.

p.2013 *Trigonosaurus pricei* Valieri and Díaz

.2014 *Baurutitan britoi* Lacovara et al.

.2014 *Baurutitan britoi* Poropat et al.

p.2014 *Trigonosaurus pricei* Salgado et al.

p.2014 *Baurutitan britoi* Saegusa and Ikeda

p.2015 *Trigonosaurus pricei* Poropat et al.

p.2015 *Trigonosaurus pricei* Zurriaguz and Powell

.2016 *Baurutitan britoi* Bandeira et al.

.2016 *Trigonosaurus pricei* Bandeira et al.

.2016 *Baurutitan britoi* D’emic et al.

.2016 *Baurutitan britoi* Dal Sasso et al.

.2016 *Trigonosaurus pricei* Dal Sasso et al.

.2016 *Baurutitan britoi* França et al.

.2016 *Baurutitan britoi* González Riga et al.

p.2016 *Trigonosaurus pricei* González Riga et al.

p.2016 *Trigonosaurus pricei* Martínez et al.

p.2016 *Trigonosaurus pricei* Ullman and Lacovara

p.2016 *Baurutitan britoi* Tykoski and Fiorillo

p.2017 *Trigonosaurus pricei* Averianov and Skutschas

.2017 *Baurutitan britoi* Carballido et al.

p.2017 *Trigonosaurus pricei* Carballido et al.

p.2017 *Trigonosaurus pricei* Fernandez-Baldor et al.

.2017 *Baurutitan britoi* Gorscak et al

p.2017 *Trigonosaurus pricei* Gorscak et al

p.2017 *Trigonosaurus pricei* Poropat et al.

p.2017 *Trigonosaurus pricei* Sassani and Bivens

p.2017 *Trigonosaurus pricei* Voegele et al.

.2018 *Baurutitan britoi* Díez Díaz et al.

.2018 *Baurutitan britoi* Hechenleitner et al.

.2018 *Baurutitan britoi* Salam et al.

.2019 *Trigonosaurus pricei* Apesteguía et al.

?2019 *Trigonosaurus pricei* Averianov et al.

.2019 *Baurutitan britoi* Averianov and Lopatin

?2019 *Trigonosaurus pricei* Averianov and Lopatin

p.2019 *Trigonosaurus pricei* Bandeira et al.

.2019 *Baurutitan britoi* Bandeira et al.

.2019 *Baurutitan britoi* Filippi et al.

p.2019 *Trigonosaurus pricei* Filippi et al.

p.2019 *Trigonosaurus pricei* González Riga et al.

.2019 *Baurutitan britoi* Gorscak and O’Connor

.2019 *Baurutitan britoi* Mannion et al.

.2019 *Trigonosaurus pricei* Novas et al.

.2020 *Baurutitan britoi* Carballido et al.

.2020 *Baurutitan britoi* Hechenleitner et al.

.2020 *Baurutitan britoi* Otero et al.

p.2020 *Trigonosaurus pricei* Poropat et al.

.2021 *Baurutitan britoi* Bellardini et al.

p.2021 *Trigonosaurus pricei* Bellardini et al.

.2021 *Baurutitan britoi* Cerda et al.

p.2021 *Trigonosaurus pricei* Cerda et al.

p.2021 *Baurutitan britoi* Pérez-Moreno et al.

.2021 *Baurutitan britoi* Otero et al.

p.2021 *Trigonosaurus pricei* Rubilar-Rogers et al.

p.2021 *Baurutitan britoi* Roland et al.

.2021 *Baurutitan britoi* Silva Junior et al.

.2021 *Baurutitan britoi* Soto et al.

**References**

Apesteguía, S., Luzuriaga, J. E. S., Gallina, P. A., Granda, J. T., & Jaramillo, G. A. G. (2020). The first dinosaur remains from the Cretaceous of Ecuador. Cretaceous Research, 108, 104345.

Averianov, A., & Skutschas, P. (2017). A new lithostrotian titanosaur (Dinosauria, Sauropoda) from the Early Cretaceous of Transbaikalia, Russia. Biological Communications, (1), 6-18.

Averianov, A. O., & Lopatin, A. V. (2019). Sauropod diversity in the Upper Cretaceous Nemegt Formation of Mongolia-a possible new specimen of *Nemegtosaurus*. Acta Palaeontologica Polonica, 64(2).

Averianov, A. O., Ivantsov, S. V., & Skutschas, P. P. (2019). Caudal vertebrae of titanosaurian sauropod dinosaurs from the Lower Cretaceous Ilek Formation in Western Siberia, Russia. Cretaceous Research, 107, 104309.

Bandeira, K. L., Medeiros Simbras, F., Batista Machado, E., de Almeida Campos, D., Oliveira, G. R., & Kellner, A. W. (2016). A new giant titanosauria (Dinosauria: Sauropoda) from the late cretaceous Bauru Group, Brazil. PloS one, 11(10), e0163373.

Bandeira, K. L., Machado, E. B., Campos, D., & Kellner, A. W. (2019). New titanosaur (Sauropoda, Dinosauria) records from the Morro do Cambambe unit (upper cretaceous), Mato Grosso state, Brazil. Cretaceous Research, 103, 104155.

Barco, J. L. (2009). Sistemática e implicaciones filogenéticas y paleobiogeográficas del saurópodo *Galvesaurus herreroi* (Formación Villar del Arzobispo, Galve, España). Unpublished PhD thesis, Universidad de Zaragoza.

Bellardini, F., Windholz, G. J., Baiano, M. A., Garrido, A. C., & Filippi, L. S. (2021). New titanosaur remains from the Portezuelo Formation (Turonian–Coniancian) and their implications for the sauropod faunal diversity of the southern Neuquén Basin, Patagonia, Argentina. Journal of South American Earth Sciences, 111, 103457.

Bonaparte, J. F., & Coria, R. A. (1993). Un nuevo y gigantesco saurópodo titanosaurio de la Formación Río Limay (Albiano-Cenomaniano) de la Provincia del Neuquén, Argentina. Ameghiniana, 30(3), 271-282.

Calvo, J. O., González Riga, B. J., & Porfiri, J. D. (2007). A new titanosaur sauropod from the Late Cretaceous of Neuquén, Patagonia, Argentina. Arquivos do Museu Nacional, 65(4), 485-504.

Casal, G., Martínez, R., Luna, M. Sciutto, J. C., & Lamanna, M. (2007). *Aeolosaurus colhuehuapensis* sp. nov. (Sauropoda, Titanosauria) de la Formación Bajo Barreal, Cretácico Superior de Argentina. Revista Brasileira de Paleontologia, 10(1), 53-62.

Carballido, J. L., & Sander, P. M. (2014). Postcranial axial skeleton of *Europasaurus holgeri* (Dinosauria, Sauropoda) from the Upper Jurassic of Germany: implications for sauropod ontogeny and phylogenetic relationships of basal Macronaria. Journal of Systematic Palaeontology, 12(3), 335-387.

Carballido, J. L., Pol, D., Otero, A., Cerda, I. A., Salgado, L., Garrido, A. C. & Krause, J. M. (2017). A new giant titanosaur sheds light on body mass evolution among sauropod dinosaurs. Proceedings of the Royal Society B: Biological Sciences, 284(1860), 20171219.

Carballido, J. L., Scheil, M., Knötschke, N., & Sander, P. M. (2020). The appendicular skeleton of the dwarf macronarian sauropod *Europasaurus holgeri* from the Late Jurassic of Germany and a re-evaluation of its systematic affinities. Journal of Systematic Palaeontology, 18(9), 739-781.

Cerda, I., Zurriaguz, V. L., Carballido, J. L., González, R., & Salgado, L. (2021). Osteology, paleohistology and phylogenetic relationships of *Pellegrinisaurus powelli* (Dinosauria: Sauropoda) from the Upper Cretaceous of Argentinean Patagonia. Cretaceous Research, 128, 104957.

Coria, R. A., Filippi, L. S., Chiappe, L. M., Garcia, R., & Arcucci, A. B. (2013). *Overosaurus paradasorum* gen. et sp. nov., a new sauropod dinosaur (Titanosauria: Lithostrotia) from the Late Cretaceous of Neuquén, Patagonia, Argentina. Zootaxa, 3683(4), 357-376.

Csiki, Z., Codrea, V., Jipa-Murzea, C., & Godefroit, P. (2010). A partial titanosaur (Sauropoda, Dinosauria) skeleton from the Maastrichtian of Nalat-Vad, Hateg Basin, Romania. Neues Jahrbuch für Geologie und Paläontologie-Abhandlungen, 297-324.

D'Emic, M., Wilson, J. A., & Williamson, T. E. A Sauropod Dinosaur Pes from the Latest Cretaceous of North America and the Validity of *Alamosaurus sanjuanensis* (Sauropoda, Titanosauria). Journal of Vertebrate Paleontology, 31(5), 1072-1079.

D'Emic, M. D., Mannion, P. D., Upchurch, P., Benson, R. B., Pang, Q., & Zhengwu, C. (2013). Osteology of *Huabeisaurus allocotus* (Sauropoda: Titanosauriformes) from the upper Cretaceous of China. PLoS One, 8(8), e69375.

Dal Sasso, C., Pierangelini, G., Famiani, F., Cau, A., & Nicosia, U. (2016). First sauropod bones from Italy offer new insights on the radiation of Titanosauria between Africa and Europe. Cretaceous Research, 64, 88-109.

Díaz, V. D., Garcia, G., Pereda-Suberbiola, X., Jentgen-Ceschino, B., Stein, K., Godefroit, P., & Valentin, X. (2018). The titanosaurian dinosaur *Atsinganosaurus velauciensis* (Sauropoda) from the Upper Cretaceous of southern France: new material, phylogenetic affinities, and palaeobiogeographical implications. Cretaceous Research, 91, 429-456.

Fernández-Baldor, F. T., Canudo, J. I., Huerta, P., Moreno-Azanza, M., & Montero, D. (2017). *Europatitan eastwoodi*, a new sauropod from the lower Cretaceous of Iberia in the initial radiation of somphospondylans in Laurasia. PeerJ, 5, e3409.

Filippi, L. S., Canudo, J. I., Salgado, J. L., Garrido, A., García, R., Cerda, I., & Otero, A. (2011). A new sauropod titanosaur from the Plottier Formation (Upper Cretaceous) of Patagonia (Argentina). Geologica Acta: an international earth science journal, 9(1), 1-12.

Filippi, L. S., Martinelli, A. G., & Garrido, A. C. (2013). Registro de un dinosaurio Aeolosaurini (Sauropoda, Titanosauria) en el Cretácico Superior (Formación Plottier) del Norte de la Provincia de Neuquén, Argentina, y comentarios sobre los Aeolosaurini sudamericanos. Revista Brasileira de Paleontologia, 16(147), e156.

Filippi, L. S., Salgado, L., & Garrido, A. C. (2019). A new giant basal titanosaur sauropod in the Upper Cretaceous (Coniacian) of the Neuquén Basin, Argentina. Cretaceous Research, 100, 61-81.

Franco-Rosas, A. C., Salgado, L. E. O. N. A. R. D. O., Rosas, C. F., & Carvalho, I. D. S. (2004). Nuevos materiales de titanosaurios (Sauropoda) en el Cretácico superior de Mato Grosso, Brasil. Revista Brasileira de Paleontologia, 7(3), 329-336.

França, M. A., Júlio, C. D. A., Riff, D., Hsiou, A. S., & Langer, M. C. (2016). New lower jaw and teeth referred to *Maxakalisaurus topai* (Titanosauria: Aeolosaurini) and their implications for the phylogeny of titanosaurid sauropods. PeerJ, 4, e2054.

Gallina, P. A. (2011). Notes on the axial skeleton of the titanosaur *Bonitasaura salgadoi* (Dinosauria-Sauropoda). Anais da Academia Brasileira de Ciências, 83(1), 235-246.

Garcia, G., Amico, S., Fournier, F., Thouand, E., & Valentin, X. (2010). A new titanosaur genus (Dinosauria, Sauropoda) from the Late Cretaceous of southern France and its paleobiogeographic implications. Bulletin de la Société géologique de France, 181(3), 269-277.

González Riga, B. J., Lamanna, M. C., Ortiz David, L. D., Calvo, J. O., & Coria, J. P. (2016). A gigantic new dinosaur from Argentina and the evolution of the sauropod hind foot. Scientific Reports, 6(1), 1-15.

González Riga, B. J., Lamanna, M. C., Otero, A., David, L. D. O., Kellner, A. W., & Ibiricu, L. M. (2019). An overview of the appendicular skeletal anatomy of South American titanosaurian sauropods, with definition of a newly recognized clade. Anais da Academia Brasileira de Ciências, 91.

Gorscak, E., O'Connor, P. M., Roberts, E. M., & Stevens, N. J. (2017). The second titanosaurian (Dinosauria: Sauropoda) from the middle Cretaceous Galula Formation, southwestern Tanzania, with remarks on African titanosaurian diversity. Journal of Vertebrate Paleontology, 37(4), e1343250.

Gorscak, E., & O’Connor, P. M. (2019). A new African Titanosaurian Sauropod Dinosaur from the middle Cretaceous Galula Formation (Mtuka Member), Rukwa Rift Basin, Southwestern Tanzania. PloS one, 14(2), e0211412.

Hechenleitner, E. M., Fiorelli, L. E., Martinelli, A. G., & Grellet-Tinner, G. (2018). Titanosaur dinosaurs from the Upper Cretaceous of La Rioja province, NW Argentina. Cretaceous Research, 85, 42-59.

Hechenleitner, E. M., Leuzinger, L., Martinelli, A. G., Rocher, S., Fiorelli, L. E., Taborda, J. R., & Salgado, L. (2020). Two Late Cretaceous sauropods reveal titanosaurian dispersal across South America. Communications biology, 3(1), 1-13.

Kellner, A. W., Campos, D. D. A., & Trotta, M. N. F. (2005). Description of a titanosaurid caudal series from the Bauru Group, Late Cretaceous of Brazil. Arquivos do Museu Nacional, 63(3), 529-564.

Lacovara, K. J., Lamanna, M. C., Ibiricu, L. M., Poole, J. C., Schroeter, E. R., Ullmann, P. V. & Novas, F. E. (2014). A gigantic, exceptionally complete titanosaurian sauropod dinosaur from southern Patagonia, Argentina. Scientific Reports, 4(1), 1-9.

Machado, E. B., Avilla, L. D. S., Nava, W. R., Campos, D. D. A., & Kellner, A. W. (2013). A new titanosaur sauropod from the Late Cretaceous of Brazil. Zootaxa, 3701(3), 301-321.

Mannion, P. D., Upchurch, P., Barnes, R. N., & Mateus, O. (2013). Osteology of the Late Jurassic Portuguese sauropod dinosaur *Lusotitan atalaiensis* (Macronaria) and the evolutionary history of basal titanosauriforms. Zoological Journal of the Linnean Society, 168(1), 98-206.

Mannion, P. D., Upchurch, P., Schwarz, D., & Wings, O. (2019). Taxonomic affinities of the putative titanosaurs from the Late Jurassic Tendaguru Formation of Tanzania: phylogenetic and biogeographic implications for eusauropod dinosaur evolution. Zoological Journal of the Linnean Society, 185(3), 784-909.

Martinelli, A. G., Riff, D., & Lopes, R. P. (2011). Discussion about the occurrence of the genus *Aeolosaurus* Powell 1987 (Dinosauria, Titanosauria) in the Upper Cretaceous of Brazil. Gaea: Journal of Geoscience, 7(1), 34.

Martínez, R. D., Lamanna, M. C., Novas, F. E., Ridgely, R. C., Casal, G. A., Martínez, J. E. & Witmer, L. M. (2016). A basal lithostrotian titanosaur (Dinosauria: Sauropoda) with a complete skull: implications for the evolution and paleobiology of Titanosauria. PloS one, 11(4), e0151661.

Matthews, S. C. (1973). Notes on open nomenclature and on synonymy lists. Palaeontology, 16(4), 713-719.

Michael, D. D., Foreman, B. Z., & Jud, N. A. (2016). Anatomy, systematics, paleoenvironment, growth, and age of the sauropod dinosaur *Sonorasaurus thompsoni* from the Cretaceous of Arizona, USA. Journal of Paleontology, 90(1), 102-132.

Moreno, A. P., Carballido, J. L., Otero, A., Salgado, L., & Calvo, J. O. (2022). The Axial Skeleton of *Rinconsaurus caudamirus* (Sauropoda: Titanosauria) from the Late Cretaceous of Patagonia, Argentina. Ameghiniana, 59(1), 1-46.

Novas, F., Agnolin, F., Rozadilla, S., Aranciaga-Rolando, A. M., Brisson-Egli, F., Motta, M. J. & Salgado, L. (2019). Paleontological discoveries in the Chorrillo Formation (upper Campanian-lower Maastrichtian, Upper Cretaceous), Santa Cruz Province, Patagonia, Argentina. Revista del Museo Argentino de Ciencias Naturales, 21(2), 217-293.

Otero, A., Carballido, J. L., Salgado, L., Canudo, J. I., & Garrido, A. C. (2020). Report of a giant titanosaur sauropod from the Upper Cretaceous of Neuquén Province, Argentina. Cretaceous Research, 122, 104754.

Otero, A., Carballido, J. L., Salgado, L., Canudo, J. I., & Garrido, A. C. (2021). Report of a giant titanosaur sauropod from the Upper Cretaceous of Neuquén Province, Argentina. Cretaceous Research, 122, 104754.

Porfiri, J., & Calvo, J. O. (2010). *Panamericansaurus schroederi* gen. nov. sp. nov. un nuevo Sauropoda (Titanosauridae-Aeolosaurini) de la Provincia del Neuquén, Cretácico Superior de Patagonia, Argentina. Brazilian Geographical Journal: Geosciences and Humanities research medium, 1(1), 4.

Poropat, S. F., Mannion, P. D., Upchurch, P., Hocknull, S. A., Kear, B. P., & Elliott, D. A. (2015). Reassessment of the non‐titanosaurian somphospondylan *Wintonotitan wattsi* (Dinosauria: Sauropoda: Titanosauriformes) from the mid‐Cretaceous Winton Formation, Queensland, Australia. Papers in Palaeontology, 1(1), 59-106.

Poropat, S. F., Nair, J. P., Syme, C. E., Mannion, P. D., Upchurch, P., Hocknull, S. A. & Holland, T. (2017). Reappraisal of *Austrosaurus mckillopi* Longman, 1933 from the Allaru Mudstone of Queensland, Australia’s first named Cretaceous sauropod dinosaur. Alcheringa: An Australasian Journal of Palaeontology, 41(4), 543-580.

Poropat, S. F., Mannion, P. D., Upchurch, P., Tischler, T. R., Sloan, T., Sinapius, G. H. & Elliott, D. A. (2020). Osteology of the wide-hipped titanosaurian sauropod dinosaur *Savannasaurus elliottorum* from the Upper Cretaceous Winton Formation of Queensland, Australia. Journal of Vertebrate Paleontology, 40(3), e1786836.

Rolando, M. A., Marsà, J. A. G., Agnolín, F. L., Motta, M. J., Rozadilla, S., & Novas, F. E. (2022). The sauropod record of Salitral Ojo del Agua: An Upper Cretaceous (Allen Formation) fossiliferous locality from northern Patagonia, Argentina. Cretaceous Research, 129, 105029.

Rubilar-Rogers, D., Vargas, A. O., Riga, B. G., Soto-Acuña, S., Alarcón-Muñoz, J., Iriarte-Díaz, J. & Gutstein, C. S. (2021). *Arackar licanantay* gen. et sp. nov. a new lithostrotian (Dinosauria, Sauropoda) from the Upper Cretaceous of the Atacama Region, northern Chile. Cretaceous Research, 124, 104802.

Sallam, H. M., Gorscak, E., O’Connor, P. M., El-Dawoudi, I. A., El-Sayed, S., Saber, S. & Lamanna, M. C. (2018). New Egyptian sauropod reveals Late Cretaceous dinosaur dispersal between Europe and Africa. Nature Ecology & Evolution, 2(3), 445-451.

Salgado, L., Coria, R. A., & Calvo, J. O. (1997). Evolution of titanosaurid sauropods: Phylogenetic analysis based on the postcranial evidence. Ameghiniana, 34(1), 3-32.

Salgado, L., García, R., & Daza, J. (2006). Consideraciones sobre las láminas neurales de los dinosaurios saurópodos y su significado morfofuncional. Revista del Museo Argentino de Ciencias Naturales nueva serie, 8(1), 69-79.

Salgado, L., & De Souza Carvalho, I. (2008). *Uberabatitan ribeiroi*, a new titanosaur from the Marília formation (Bauru group, Upper Cretaceous), Minas Gerais, Brazil. Palaeontology, 51(4), 881-901.

Salgado, L., Gallina, P. A., & Paulina Carabajal, A. (2015). Redescription of *Bonatitan reigi* (Sauropoda: Titanosauria), from the Campanian–Maastrichtian of the Río Negro Province (Argentina). Historical Biology, 27(5), 525-548.

Santucci, R. M., & Bertini, R. J. (2006). A new titanosaur from western São Paulo state, upper Cretaceous Bauru Group, south‐east Brazil. Palaeontology, 49(1), 59-66.

Santucci, R. M., & Arruda-Campos, A. D. (2011). A new sauropod (Macronaria, Titanosauria) from the Adamantina Formation, Bauru Group, Upper Cretaceous of Brazil and the phylogenetic relationships of Aeolosaurini. Zootaxa, 3085(1), 1-33.

Sassani, N., & Bivens, G. T. (2017). The Chinese colossus: an evaluation of the phylogeny of *Ruyangosaurus giganteus* and its implications for titanosaur evolution (No. e2988v1). PeerJ Preprints.

Silva Junior, J. C., Martinelli, A. G., Iori, F. V., Marinho, T. S., Hechenleitner, E. M., & Langer, M. C. (2022). Reassessment of *Aeolosaurus maximus*, a titanosaur dinosaur from the Late Cretaceous of Southeastern Brazil. Historical Biology, 34(3), 403-411.

Soto, M., Montenegro, F., Mesa, V., & Perea, D. (2022). Sauropod (Dinosauria: Saurischia) remains from the Mercedes and Asencio formations (sensu Bossi, 1966), Upper Cretaceous of Uruguay. Cretaceous Research, 131, 105072.

Tykoski, R. S., & Fiorillo, A. R. (2017). An articulated cervical series of *Alamosaurus sanjuanensis* Gilmore, 1922 (Dinosauria, Sauropoda) from Texas: new perspective on the relationships of North America's last giant sauropod. Journal of Systematic Palaeontology, 15(5), 339-364.

Ullmann, P. V., & Lacovara, K. J. (2016). Appendicular osteology of *Dreadnoughtus schrani*, a giant titanosaurian (Sauropoda, Titanosauria) from the Upper Cretaceous of Patagonia, Argentina. Journal of Vertebrate Paleontology, 36(6), e1225303.

Valieri, R. J., & Díaz, S. D. R. (2013). Assignation of the vertebra CPP 494 to *Trigonosaurus pricei Campos* et al., 2005 (Sauropoda: Titanosauriformes) from the Late Cretaceous of Brazil, with comments on the laminar variation among lithostrotian titanosaurs. Boletín del Museo Nacional de Historia Natural del Paraguay, 17(1), 20-28.

Voegele, K., Lamanna, M., & Lacovara, K. (2017). Osteology of the dorsal vertebrae of the giant titanosaurian sauropod dinosaur *Dreadnoughtus schrani* from the Late Cretaceous of Argentina. Acta Palaeontologica Polonica, 62.

Wilson, J. A., & Sereno, P. C. (1998). Early evolution and higher-level phylogeny of sauropod dinosaurs. Journal of vertebrate paleontology, 18(S2), 1-79.

Zurriaguz, V., & Powell, J. (2015). New contributions to the presacral osteology of *Saltasaurus loricatus* (Sauropoda, Titanosauria) from the Upper Cretaceous of northern Argentina. Cretaceous Research, 54, 283-300.

**APPENDIX III. Scorings for the phylogenetic analyses.**

**Iteration I**

*Baurutitan_britoi* ??????????????????????????????????????????????????????????????????????????????????????????????????????????????????????????????????????????????????????????????????????????????????????????????????????????-??????????????????02220001030(12)001110-00200100000310002101100???0-111?????????????????????????????????????????????????????????????????????????????????????????????????????????????????????????????????????????00001????1010?0000

BR-262 ?????????????????????????????????????????????????????????????????????????????????????????????????????????????????????????11013?0?00201020?0021000?1????????11033310121?1122100122(01)120110201010?11110110111-0?(01)221?1??????????0????001030(12)001110-00200100000310002101100???0??????1113000010011?????11101?01?10100120?????????????????????????????0001??11000????????????????????????????????????????????????????????????00001????1010?0000

*Trigonosaurus_pricei* ?????????????????????????????????????????????????????????????????????????????????????????????????????????????????????????11013?0?00301020?00210000111?100??11033310121?1122100122(01)120110201010?11120110111?0?(0 1)221?1??31?010???????????????????????????????????????????????????????????????????????????????????????????????????????????????????????????????????????????????????????????????????????????????????????????????????????????????

*Caieiria_allocaudata* ??????????????????????????????????????????????????????????????????????????????????????????????????????????????????????????????????????????????????????????????????????????????????????????????????????????-??????????????????00???0010301000110-00200100030310001?00????????????????????????????????????????????????????????????????????????????????????????????????????????????????????????????????????????????????????00001?????020?0000

**Iteration II**

*Baurutitan_britoi* ?????????????????????????????????????????????????????????????????????????????????????????????????????????????????????????11013?0?00301020?00210000111?100??11033310121?1122100122(01)120110201010?111(12)0110111-0?(01)221?1??31?010??02220001030(12)001110-00200100000310002101100???0-111??1113000010011?????11101?01?10100120?????????????????????????????0001??11000????????????????????????????????????????????????????????????00001????1010?0000

*Caieiria_allocaudata* ??????????????????????????????????????????????????????????????????????????????????????????????????????????????????????????????????????????????????????????????????????????????????????????????????????????-??????????????????00???0010301000110-00200100030310001?00????????????????????????????????????????????????????????????????????????????????????????????????????????????????????????????????????????????????????00001?????020?0000
