# Supplementary material for: New specimens of Baurutitan britoi and a taxonomic reassessment of the titanosaur dinosaur fauna (Sauropoda) from the Serra da Galga Formation (Late Cretaceous) of Brazil
Source: PeerJ. 2022 Nov 15;10:e14333. doi: 10.7717/peerj.14333 (PMC9673870; doi:10.7717/peerj.14333)
Supplement: Supplemental Information 3 — Signed permission to use a third party image. [file peerj-10-14333-s003.pdf]

PeerJ Permission Letter

All items (1-5) are required:

1. Title of PeerJ submission: **New specimens of Baurutitan britoi and a taxonomic reassessment of the titanosaur dinosaur fauna (Sauropoda) from the Serra da Galga Formation (Late Cretaceous) of Brazil**
2. Printed name of copyright holder: **Beethoven Teixeira**
3. Select copyrighted item: photographs | videos | other (please describe): **Scanned version of a newspaper article**
4. *For figures*: Supply the approved images(s) including the figure number(s) as they appear in the PeerJ submission OR A complete description of the image(s) : **Image 1.**

B

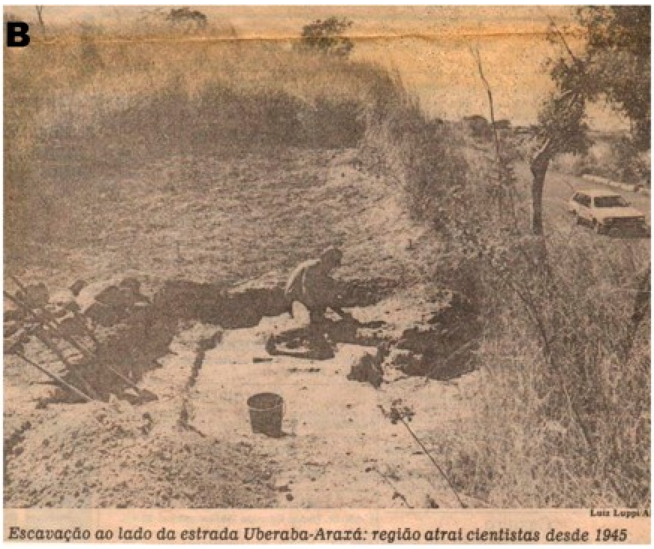

Escavação ao lado da estrada Uberaba-Araxá: região atrai cientistas desde 1945

C

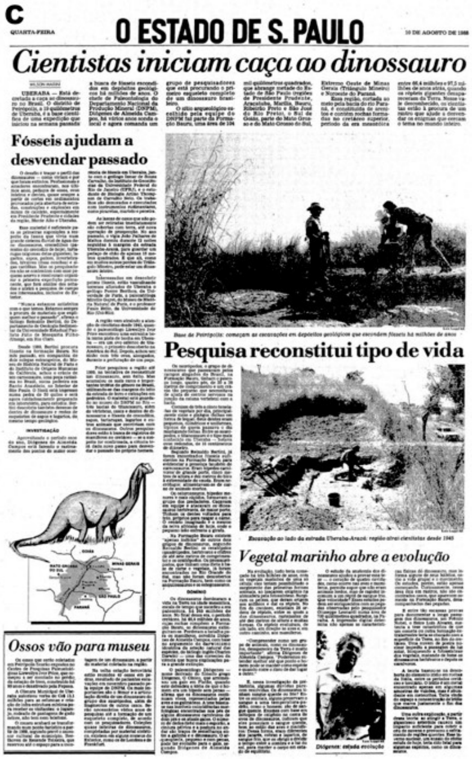

QUARTA-FEIRA

O ESTADO DE S. PAULO

Cientistas iniciam caça ao dinossauro

Fósseis ajudam a desvendar passado

Pesquisa reconstitui tipo de vida

Vegetal marinho abre a evolução

Ossos vão para museu

5. I give my permission to PeerJ to publish my work, as described and/or appear below, under the CC-BY 4.0 license.

Signature of copyright holder

October 4, 2019
